# Nephroprotective Potential of *Olea europaea* Leaf Extract Against Combined Noise and Toluene‐Induced Oxidative and Histopathological Renal Injuries in Wistar Rats

**DOI:** 10.1002/cbdv.202502857

**Published:** 2025-11-05

**Authors:** Takoua Ben Attia, Mabrouk Horchani, Mariem Salhi, Zeineb Dhifallah, Meriem Ksentini, Hichem Ben Jannet, Abada Mhamdi

**Affiliations:** ^1^ Laboratory of Research “LR 23/ES/10”: Population Pathology, Environmental Aggressors, Alternative Therapy, Faculty of Medicine of Tunis University of Tunis El Manar Tunis Tunisia; ^2^ Laboratory of Heterocyclic Chemistry, Natural Products and Reactivity (LR11Es39), Medicinal Chemistry and Natural Products, Faculty of Science of Monastir University of Monastir Monastir Tunisia; ^3^ Westlake Laboratory of Life Sciences and Biomedicine Institute of Basic Medical Sciences Westlake Institute for Advanced Study, School of Life Sciences Westlake University Hangzhou China; ^4^ Research Laboratory LR23ES02 “Theranostic Biomarkers” Pathology Department Charles Nicolle University Hospital University of Tunis El Manar Tunis Tunisia

**Keywords:** kidney, nephroprotection, noise, *Olea europaea* leaf extract, oxidative stress, toluene

## Abstract

Noise and toluene are environmental stressors that induce oxidative stress and inflammation, contributing to renal dysfunction. This study evaluated the protective effects of *Olea europaea* leaf extract (OeLE; 40 mg/kg/day) against combined noise‐ and toluene‐induced nephrotoxicity in Wistar rats. Forty‐eight male rats were divided into eight groups and exposed for 6 weeks (6 h/day, 5 days/week) to noise, toluene, or their combination, with or without OeLE supplementation. Renal function biomarkers (urea and creatinine), oxidative stress indices (malondialdehyde, catalase [CAT], and superoxide dismutase [SOD]), histopathology, and in silico docking targeting key redox and inflammatory mediators (CAT, SOD, IL‐1β, TNF‐α, and NF‐κB) were assessed. Co‐exposure caused significant renal dysfunction, elevated lipid peroxidation, impaired antioxidant defenses, glomerular collapse, tubular necrosis, vascular congestion, and interstitial inflammation, with the combined exposure group showing the most severe lesions. OeLE supplementation markedly mitigated these effects by restoring renal biomarkers, reducing oxidative injury, enhancing enzymatic antioxidant activity, and preserving renal architecture. Molecular docking confirmed strong interactions between OeLE bioactive compounds (oleuropein and luteolin‐7‐O‐glucoside) and antioxidant/inflammatory mediators. OeLE exerts antioxidant and anti‐inflammatory effects, protecting the kidneys from combined noise‐ and toluene‐induced damage, highlighting its potential as a natural therapeutic agent.

## Graphical abstract

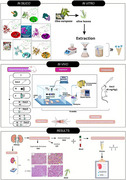

## 1 Introduction

For centuries, traditional medicine has relied on the therapeutic virtues of medicinal plants as natural remedies for preventing and treating various diseases. These plants, rich in bioactive compounds, continue to serve as valuable sources of pharmacologically active agents, inspiring contemporary research in drug discovery and integrative medicine.

The olive tree (*Olea europaea*), a longstanding emblem of resilience and vitality across the Mediterranean Basin, is prized not only for its fruits and oil but also for the therapeutic properties of its leaves. In Tunisia, where olive cultivation is deeply rooted in both cultural heritage and the national economy, olive leaves have traditionally been employed for their medicinal benefits [1]. These leaves are rich in bioactive phytochemicals, notably polyphenols, flavonoids, secoiridoids, and triterpenes. Among them, oleuropein and hydroxytyrosol have garnered particular attention because of their potent antioxidant, anti‐inflammatory, and antimicrobial activities [2, 3]. These compounds play crucial roles in scavenging free radicals, mitigating oxidative stress, and modulating the key inflammatory pathways. Scientific evidence supports that olive leaf extracts can enhance endogenous antioxidant defenses, regulate cellular metabolism, and exert protective effects against a range of pathologies, particularly those associated with oxidative injury in vital organs, such as the kidneys [4, 5, 6].

In recent years, there has been growing concern regarding the impact of environmental stressors on human and animal health, particularly because of their capacity to induce systemic oxidative stress and organ‐specific toxicity. Among these stressors, chronic exposure to noise pollution and volatile organic solvents, such as toluene, has garnered increasing attention owing to their widespread prevalence in both occupational and urban environments [7, 8]. Noise, once regarded primarily as a nuisance, is now recognized as a potent biological stressor that can disrupt systemic homeostasis. Prolonged exposure to high‐intensity noise activates the hypothalamic–pituitary–adrenal (HPA) axis, elevates circulating glucocorticoids, and stimulates excessive production of reactive oxygen species (ROS). These neuroendocrine and redox disturbances contribute to vascular dysfunction, chronic inflammation, and oxidative damage in several organ systems, notably the cardiovascular, nervous, and renal systems [9, 10, 11].

Toluene, a lipophilic solvent extensively used in industrial settings and consumer products, poses substantial toxicological risks. Upon inhalation or dermal absorption, toluene is rapidly distributed to lipid‐rich tissues, including the brain and kidney [12]. In the kidneys, repeated exposure to toluene has been associated with proximal tubular degeneration, glomerular alterations, and significant disturbances in renal function biomarkers [13].

Importantly, co‐exposure to noise and toluene may exert synergistic toxicodynamic effects, wherein each stressor amplifies the deleterious effects of the other. Their combined action can intensify oxidative stress, impair endogenous antioxidant defense systems, and aggravate histopathological alterations, particularly in organs such as the heart, lungs, and liver [14, 15, 16, 17]. This organ is especially vulnerable because of its high mitochondrial density, substantial oxygen consumption, and critical role in xenobiotic elimination. In this context, sustained ROS generation can activate redox‐sensitive transcription factors, trigger proinflammatory cascades, and compromise nephron integrity. Although the individual nephrotoxic effects of noise and toluene have been documented, their interactive effects on renal physiology remain poorly understood. This constitutes a critical gap in current environmental health research, especially considering the likelihood of simultaneous exposure in real‐world scenarios.

This study aimed, for the first time, to evaluate the protective effect of olive leaf extract against renal oxidative and histopathological damage induced by combined noise and toluene exposure. The investigation integrated in vivo experimentation in Wistar rats, in silico analysis of bioactive compound–target interactions, and quantification of proinflammatory cytokines (tumor necrosis factor‐alpha [TNF‐α], interleukin‐1β [IL‐1β], and IL‐6), oxidative stress markers, and renal function biomarkers (creatinine and urea). Histological assessment provides structural insights into tissue injury.

## 2 Methods

### 2.1 Plant Material Collection, Authentication, and Extract Preparation

In November 2020, *O. europaea* L. leaves were harvested from the Siliana region near El Mansoura, northern Tunisia. To ensure precise botanical identification, a voucher sample (TBA 001) was identified and deposited in the herbarium of the Botany Department at the Faculty of Sciences of Tunis, with taxonomical confirmation by Dr. Faouzi HORCHANI.

The collected leaves were air‐dried at ambient temperature for 14 days and protected from direct sunlight to preserve their bioactive compounds. The leaves were then dried and ground into a fine powder. Five grams of this powder was extracted in 200 mL of a hydroalcoholic solution (70% ethanol and 30% water) at 60°C for 4 h. The extract was then filtered through a cellulose filter and concentrated under vacuum at 65°C using a rotary evaporator. The final concentrated extract was stored at 4°C until use. Before administration, the extract was dissolved in saline solution and delivered orally to the experimental animals via gavage, with dosages adjusted based on body weight. The extraction process was conducted at the Quality Control Laboratory of Herbes de Tunisie, located in El Mansoura, Siliana, Tunisia.

Phenolic and flavonoid contents, as well as antioxidant and anti‐inflammatory activities, were quantified following established protocols described by Ben Attia et al. [16] (data not shown).

### 2.2 In Silico Study

#### 2.2.1 Insight Into Molecular Targets Used for In Silico Study

To investigate the potential antioxidant and anti‐inflammatory mechanisms underlying the nephroprotective effects of *O. europaea* leaf extract (OeLE), we selected molecular targets based on their central roles in redox regulation and inflammatory signaling. Catalase (CAT) and superoxide dismutase (SOD) are essential antioxidant enzymes that form the first line of defense against oxidative damage by detoxifying hydrogen peroxide and superoxide radicals [18]. TNF‐α and IL‐1β are key proinflammatory cytokines that promote leukocyte recruitment, cytokine cascades, and tissue injury [19]. Nuclear factor kappa B (NF‐κB) acts as a master transcription factor that regulates the genes involved in oxidative stress and inflammation and is frequently activated under oxidative conditions [20]. Collectively, this molecular panel enables targeted evaluation of the oxidative and inflammatory components implicated in renal injury. We hypothesize that bioactive compounds from OeLE may confer nephroprotection by interacting with and modulating these critical molecular targets, thereby attenuating oxidative damage and suppressing proinflammatory pathways.

#### 2.2.2 Molecular Docking Procedure

Docking studies were performed using the AutoDock 4.2 software suite [21] to predict the molecular interactions between the bioactive compounds of OeLE and selected protein targets. The main phytochemicals investigated, including hydroxytyrosol glucoside, hydroxytyrosol, secologanoside, oleuropein aglycone, hydroxytyrosol acetate, luteolin‐7‐O‐β‐d‐glucopyranoside, and oleuropein, were identified by HPLC analysis (data not shown) and subjected to geometric optimization and energy minimization using the ACD/3D Viewer platform (http://www.filefacts.com/acd3d‐viewer‐freeware‐info). The high‐resolution crystallographic structures of the protein targets, CAT (PDB ID: 1TGU), SOD (PDB ID: 4A7G), IL‐1β (PDB ID: 6Y8M), TNF‐α (PDB ID: 2AZ5), and NF‐κB (PDB ID: 1NFK), were obtained from the RCSB Protein Data Bank (https://www.rcsb.org/). Protein preparation included the addition of missing hydrogen atoms, assignment of appropriate protonation states, and calculation of Gasteiger charges to reflect physiological conditions. Ligands and receptors were converted into PDBQT format using AutoDock Tools to ensure compatibility with the docking protocol. A grid box centered on the active site of each protein was defined with a grid‐point spacing of 0.375 Å to optimize the balance between accuracy and computational demand. The docking results were visualized and analyzed using Discovery Studio Visualizer 2017R2 (https://www.3dsbiovia.com/products/collaborative‐science/biovia‐discovery‐studio/), allowing detailed inspection of key molecular interactions, such as hydrogen bonding and hydrophobic contacts.

### 2.3 In Vivo Protocol

#### 2.3.1 Exposure System

**FIGURE 1:**
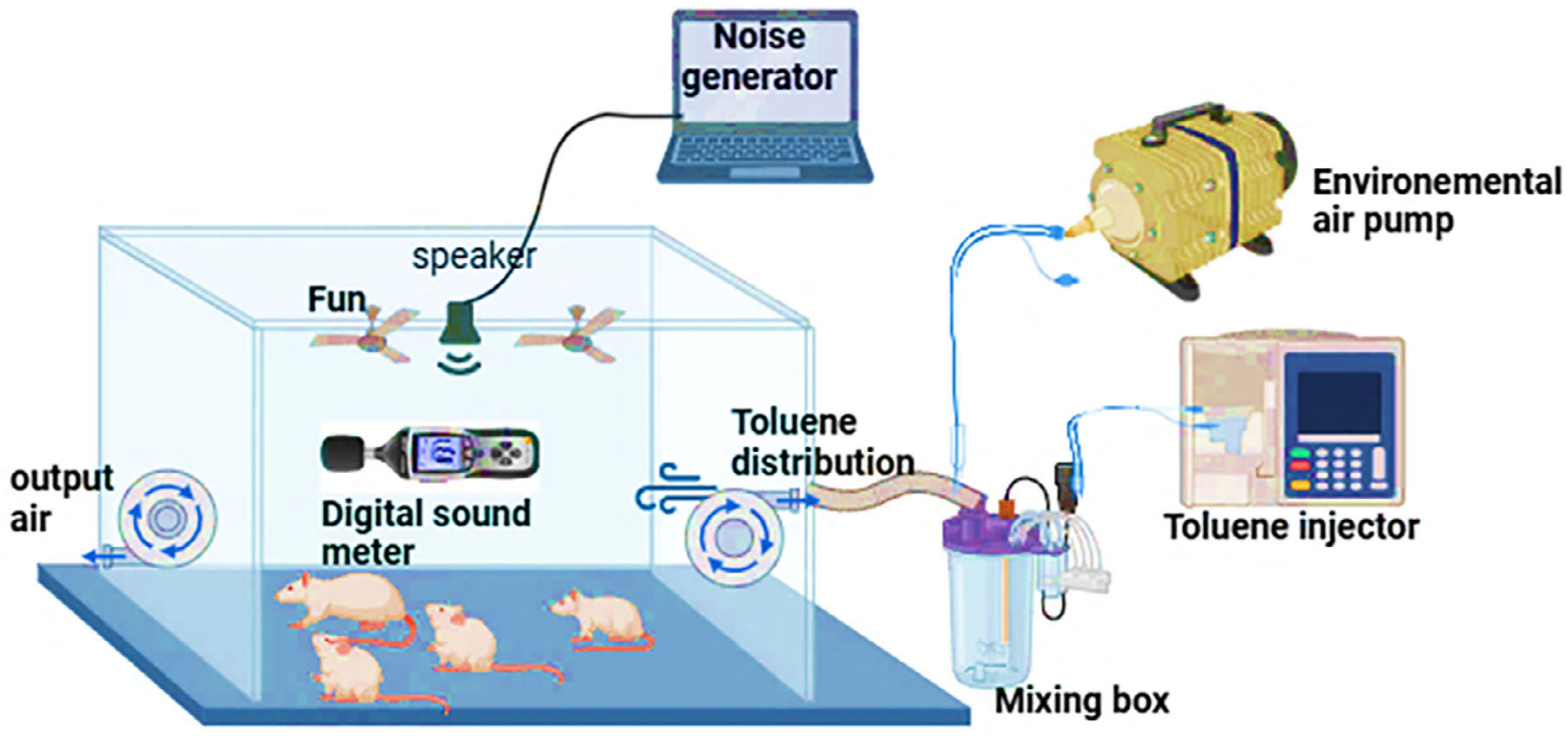
Schematic diagram of the chamber used for rat exposure to noise and toluene. Figure adapted from Ben Attia et al. [24] with permission obtained from the author.

Following the established protocol described in our recent study by Ben Attia et al. [16], rats were subjected to daily exposure to noise and/or inhaled toluene for 6 weeks (5 days/week) from 8:00 a.m. to 2:00 p.m. This exposure was conducted within specially designed Plexiglas chambers, incorporating a generation system, an exposure system, and a monitoring system (Figure 1), as detailed by Hinners et al. [22].

The acoustic exposure system was established using the Audacity 2.3.2 audio software, where the noise level was consistently set at 85 dB (A) to deliver an octave‐band noise (8–16 kHz) through a sound speaker. This speaker was uniformly positioned to ensure that all rats were subjected to the same intensity of noise. The noise level was meticulously monitored with an integration sound level meter with Class 1 accuracy, Type 2238 Bruel and Kjaer, ensuring precise exposure as per the established protocol [23].

The toluene exposure system was specifically engineered to deliver a consistent atmospheric concentration of 300 ± 10 ppm for 6 h/day within a custom‐built exposure chamber. Toluene vapor was generated by injecting liquid toluene (SMSBio, Sud Médical Services, Tunisia) into a sealed mixing vessel using an isocratic pump. The resulting vapor was conveyed into the chamber, where the airflow was regulated by a fan at the inlet and an adjustable centrifugal exhaust fan at the outlet, ensuring a homogeneous gas distribution throughout the space. Before the beginning of the experimental protocol, calibration trials were conducted to accurately adjust the system to the target concentrations. These initial verifications were performed using gas chromatography–mass spectrometry (GC–MS; Agilent Technologies 6890 N Network GC System) to establish a precise baseline for exposure. To ensure continued system reliability, weekly atmospheric quantification was performed using active air sampling. Ambient air was drawn through activated charcoal sorbent tubes using a calibrated personal air sampling pump (Casella CEL, Apex 182180B‐K5), followed by toluene desorption in carbon disulfide and GC–MS analysis. This approach enabled sensitive and traceable measurements of toluene concentrations across the exposure period. Simultaneously, real‐time monitoring was performed daily using a portable gas detection device (Dräger X‐am 8000). Manual measurements were systematically taken at three time points—before the start, at the midpoint, and at the end of each exposure session—to verify atmospheric stability and to detect any deviations in real time. The exposure chamber was equipped with circular openings to facilitate air sampling and observations. Environmental temperature was maintained at 23 ± 1°C using a digital thermometer to prevent thermal fluctuations that could interfere with vapor behavior or animal physiology.

To explore the interactive effects of noise and toluene, a co‐exposure protocol was implemented based on the operational parameters established for the individual exposure systems. In this integrated setup, both stressors were applied simultaneously within the same exposure chamber, allowing for a controlled assessment of their combined effects on the test subjects. A schematic representation of the co‐exposure chamber configuration is shown in Figure 1.

The exposure chamber, constructed from plexiglass, has an internal volume of 0.43 m^3^. Six rats were introduced into the chamber daily, where they were subjected to a 6‐h exposure protocol from 8:00 a.m. to 2:00 p.m.

#### 2.3.2 Animal Handling and Exposure Design

In this study, 48 male Wistar rats (180–200 g, aged 2–3 months) were obtained from the Pasteur Institute of Tunis and randomly assigned to eight groups of six animals each. To minimize stress, rats were housed two per cage and provided with a commercial pellet diet (Industrial Society of Food, Sfax, Tunisia) and water ad libitum. Animals were maintained under tightly controlled environmental conditions, including a 12‐h light/dark cycle and room temperature maintained at 23 ± 1°C. All experimental procedures adhered to the guidelines for the Care and Use of Animals and were approved by the Ethics Committee of the National School of Veterinary Medicine of Sidi Thabet (Ref: 162020/FMT), in accordance with the recommendations of the International Council for Laboratory Animal Science (ICLAS).

Before the exposure period, all the animals were housed under standard conditions. During the 6‐week experimental phase, exposures were conducted 5 days/week, 6 h/day (from 8:00 a.m. to 2:00 p.m.). Each day, the rats were transferred to specific exposure chambers according to their assigned groups: noise exposure (N and N + OeLE), toluene exposure (T and T + OeLE), combined noise and toluene co‐exposure (NT and NT + OeLE), or control. The control and OLE‐only groups (C and OeLE) were housed in separate chambers with background noise levels maintained below 40 ± 5 dB(A) and free from toluene exposure, ensuring environmental consistency comparable to that of the other groups.

The groups were as follows:

*Group C*: Control rats were maintained under normal conditions without exposure or treatment.

*Group OeLE*: Rats administered OeLE alone at 40 mg/kg/day for 6 weeks, administered daily at 7:00 a.m.

*Group N*: Rats exposed to noise only.

*Group N + OeLE*: Rats exposed to noise and treated with OeLE at 40 mg/kg/day for 6 weeks, administered daily at 7:00 a.m.

*Group T*: Rats exposed to toluene only.

*Group T + OeLE*: Rats exposed to toluene and treated with OeLE at 40 mg/kg/day for 6 weeks, administered daily at 7:00 a.m.

*Group NT*: Rats co‐exposed to both noise and toluene.

*Group NT + OeLE*: Rats co‐exposed to noise and toluene and treated with OeLE at 40 mg/kg/day for 6 weeks, administered daily at 7:00 a.m.

#### 2.3.3 Sample Collection

At the end of the experiment, the rats were anesthetized with ketamine chlorhydrate (50 mg/kg) and euthanized by decapitation. Blood samples were collected in heparinized tubes for biochemical plasma analysis, centrifuged at 3000 × *g* for 10 min at 4°C, and stored. The kidneys were promptly excised, rinsed with 0.1 M phosphate buffer saline (pH 6.8), and bisected. One portion was fixed in 10% neutral formalin for subsequent histological analysis, while the other was stored at −80°C for biochemical analysis.

#### 2.3.4 Biochemical Assays in Plasma

Biochemical analyses of renal function parameters, including urea and creatinine, were conducted on plasma samples. The assays were performed using the COBAS INTEGRA 400 Plus automated analyzer (Switzerland), following standardized protocols to ensure accuracy and reliability.

#### 2.3.5 Oxidative Stress Markers Determination

A portion of the kidney was weighed and washed with ice‐cold saline immediately and kept at −80°C until analysis. The kidney tissue was homogenized (ULTRA‐TURRAX IKA‐WERK, Germany) in ice‐cold PBS buffer (pH 7.2) and centrifuged for 20 min (10 000 rpm at 4°C). The supernatants were collected and maintained at −80°C until subsequent biochemical analysis.

##### 2.3.5.1 Lipid Peroxidation Determination

The determination of lipid peroxidation (LPO) was based on the detection of malondialdehyde (MDA), which was determined using the Buege and Aust [25] method. Samples were mixed with a BHT‐TCA solution (1%–20%, v/v) and centrifuged at 1000 rpm for 10 min. The supernatant was blended with TBA‐Tris solution (120–26 mM, v/v) in an acidic solution (0.6 M HCl). The mixture was heated at 100°C for 15 min and then cooled at room temperature. Absorbance was measured at 532 nm using a UV‐spectrophotometer (UV‐2650, Labomed Inc., USA) and expressed in nmol of MDA/mg of protein.

##### 2.3.5.2 CAT activity

CAT activity was measured according to the method of Aebi et al. [26]. This assay is based on the ability of this enzyme to degrade hydrogen peroxide (H_2_O_2_), which results in a decrease in the absorption of the reaction mixture at 240 nm. CAT activity was expressed in µmol/min/mg of protein.

##### 2.3.5.3 SOD activity

SOD activity was evaluated as described by Beyer and Fridovich [27] using epinephrine (50 mM carbonate buffer, pH 10.2) and bovine CAT (0.4 U/mL). This method is based on the inhibition of the auto‐oxidation of epinephrine to adrenochrome in the presence of SOD. Absorbance was measured at 480 nm, and SOD activity was expressed in USOD/min/mg of protein.

##### 2.3.5.4 Total Protein Level Determination

The protein concentration in lung samples was determined using the Bradford [28] method with bovine serum albumin (BSA) as the standard. Absorbance readings were measured at 595 nm, and the total protein content was calculated and reported in mg/mL.

#### 2.3.6 Histological Study

For histological examination, rat kidneys were perfused and immersed in a fixative solution (10% neutral buffered formalin) for 48 h, dehydrated through a graded series of ethanol, embedded in paraffin, sectioned with a microtome (Leica Biosystems RM2245, Great Britain) to obtain 4‐ to 5‐µm‐thick paraffin sections, and subsequently stained with hematoxylin and eosin (H&E).

#### 2.3.7 Statistical Analyses

Results were expressed as mean ± SD. Statistical analyses were performed using Biostat software for Windows. Significant differences between all groups (*n* = 6) were determined using the Kruskal–Wallis test followed by the Mann–Whitney *U* test for multiple comparisons with a statistical significance of *p* < 0.05.

## 3 Results

### 3.1 Phytochemical Composition and Antioxidant Capacity of OeLE

The *O. europaea* L. leaf extract (OeLE) used in this study was characterized by its phytochemical content and antioxidant activity in a previous study [16]. Quantification assays revealed high total polyphenol (85.24 mg GAE/g) and flavonoid (71.28 mg QE/g) content. The antioxidant potential, assessed via the DPPH radical scavenging assay, yielded an IC_50_ value of 13.5 µg/mL, indicating strong free radical neutralizing activity. HPLC analysis identified seven phenolic compounds: hydroxytyrosol, hydroxytyrosol glucoside, oleuropein, secologanoside, hydroxytyrosol acetate, oleuropein aglycone, and luteolin (data not shown).

The OeLE used in this study demonstrated a high content of bioactive polyphenols (85.24 mg GAE/g) and flavonoids (71.28 mg QE/g), which are key contributors to its potent antioxidant capacity (data not shown).

### 3.2 In Silico Evaluation of Bioactive Phytochemicals From OeLE

Molecular docking simulations were performed on the identified phytochemicals (hydroxytyrosol glucoside, hydroxytyrosol, secologanoside, oleuropein aglycone, hydroxytyrosol acetate, luteolin‐7‐O‐β‐d‐glucopyranoside, and oleuropein) targeting five selected molecular targets to assess their binding affinities and interaction profiles. The findings are summarized in Table 1.

**TABLE 1:** Docking binding energies (kcal/mol) of the docked ligands against the selected enzymes.

Ligand	Target receptor									
	Catalase (PDB: 1TGU)		SOD (PDB: 4A7G)		IL‐1β (PDB: 6Y8M)		TNF‐α (PDB: 2AZ5)		NF‐κB (PDB: 1NFK)	
	E*	E*	E*	E*	E*	H*	E*	H*	E*	H*
Hydroxytyrosol glucoside	−7.6	−6.1	−5.5	−6.6	−6.6	3	−6.6	2	−5.5	3
Hydroxytyrosol	−6.9	−4.8	−4.2	−5.5	−5.5	3	−5.5	1	−4.0	3
Secologanoside	−5.7	−5.0	−5.3	−6.7	−6.7	4	−6.7	4	−5.2	4
Oleuropein aglycon	−7.9	−5.2	−5.1	−6.9	−6.9	3	−6.9	4	−4.5	3
Hydroxytyrosol acetate	−7.5	−4.6	−4.2	−5.4	−5.4	0	−5.4	3	−4.6	0
Luteoline‐7‐O‐β‐d‐glucopyranoside	−9.0	−6.9	−6.4	−8.1	−8.1	4	−8.1	3	−6.1	4
Oleuropein	−8.2	−6.1	−5.8	−7.3	−7.3	4	−7.3	3	−5.7	4
Co‐crystallized ligand	−7.7	−5.1	−4.0	−8.1	−8.1	—	−8.1	0	—	—

Note: E*, binding energies (kcal/mol); H*, number of hydrogen bonds formed.

**FIGURE 2:**
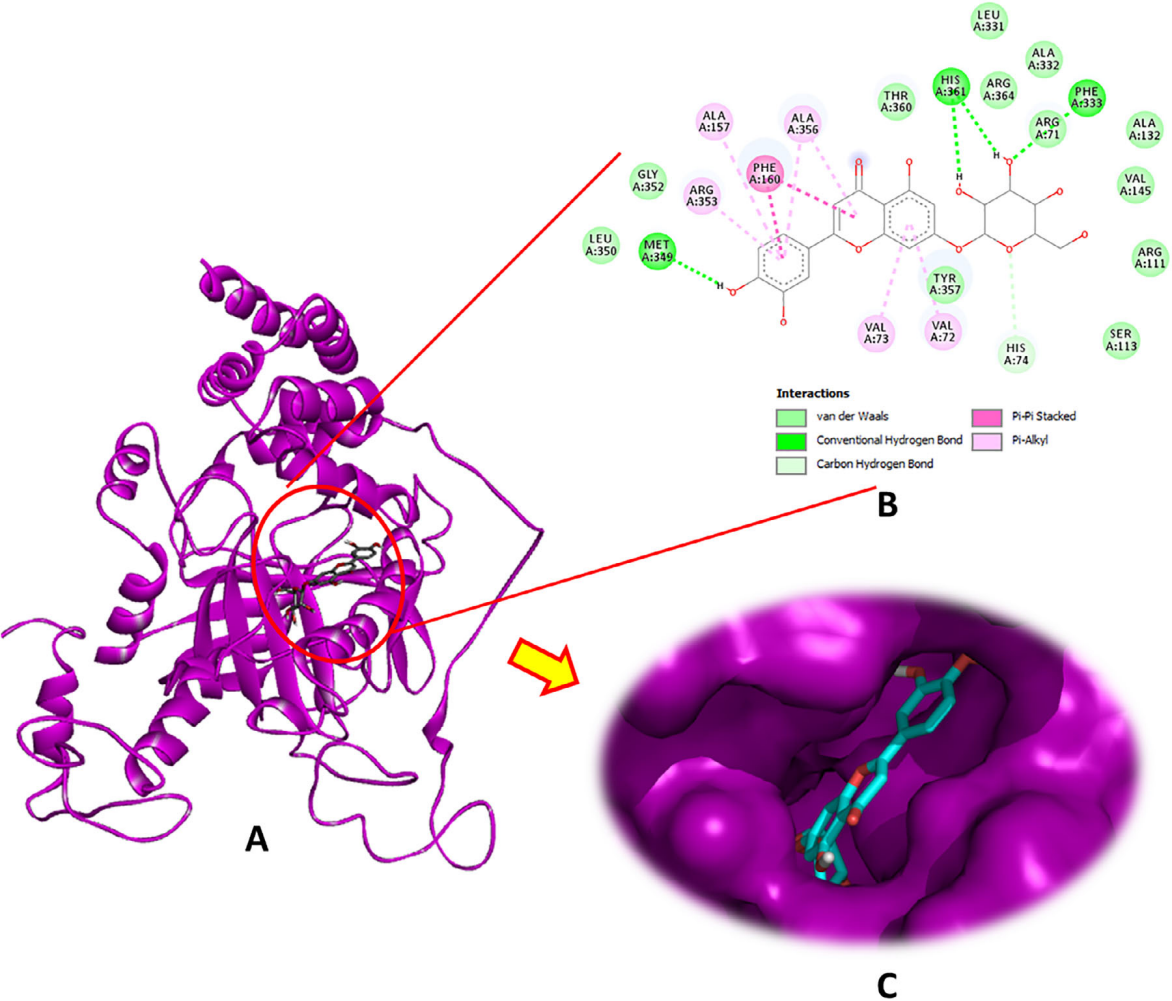
(A) Docking complex of the most bioactive compound “luteoline‐7‐O‐β‐d‐glucopyranoside” within the active site of catalase (PDB: 1TGU), (B) 2D, and (C) 3D diagrams.

Luteolin‐7‐O‐β‐d‐glucopyranoside exhibited the highest binding affinity for CAT, achieving a docking score of −9.0 kcal/mol. This compound formed four conventional hydrogen bonds through its hydroxyl groups with the crucial amino acid residues Phe333, Met349, and His361, in addition to a carbon–hydrogen bond with His74, pi–pi stacking with Phe160, and several pi–alkyl interactions involving Val72, Val723, Ala157, Arg353, and Ala356 (Figure 2). The other phytochemicals also showed significant binding interactions with CAT, suggesting potential modulatory effects (Figures S1 and S2).

**FIGURE 3:**
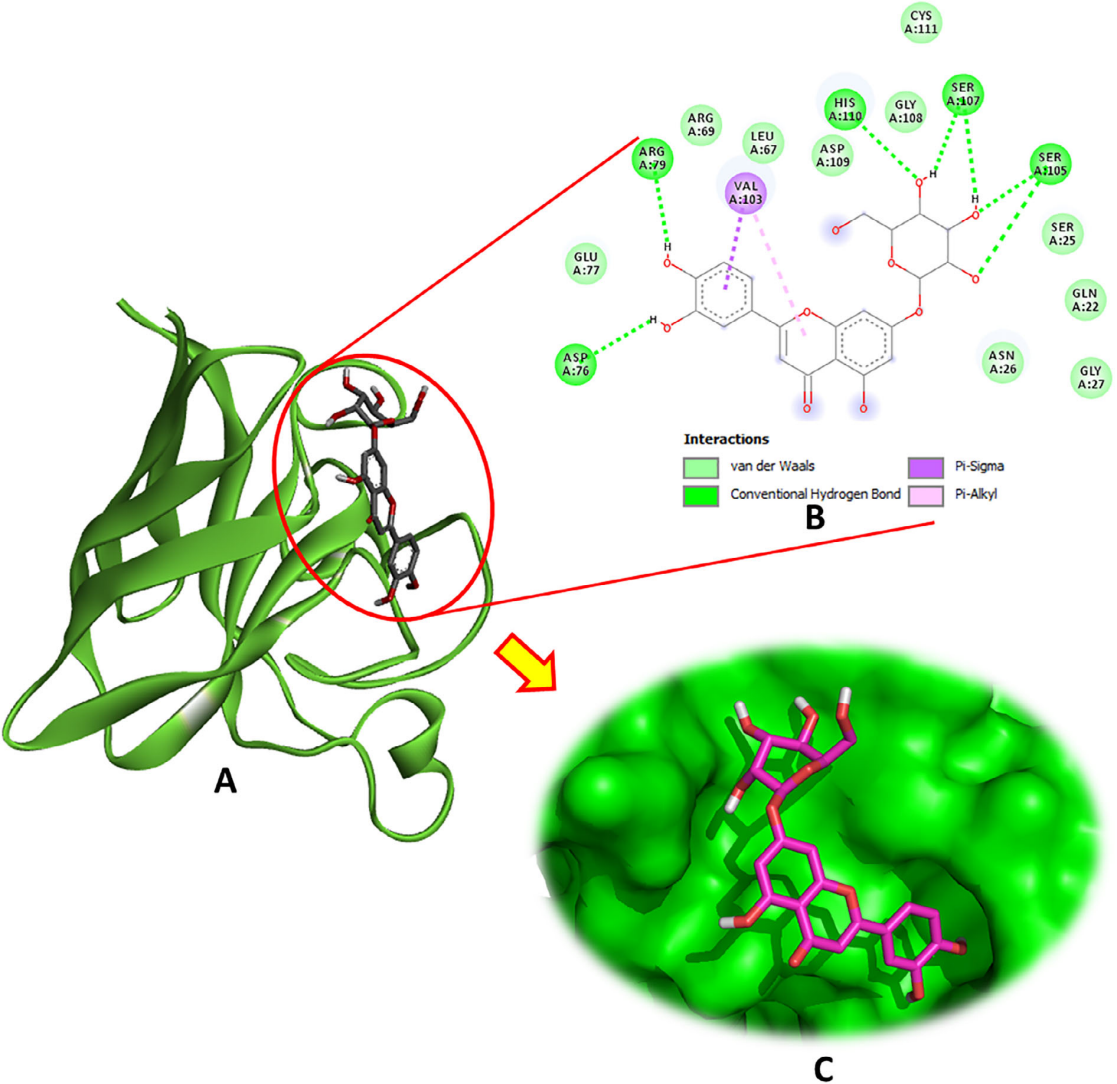
(A) Docking complex of the most bioactive compound “luteoline‐7‐O‐β‐d‐glucopyranoside” within the active site of SOD (PDB: 4A7G), (B) 2D, and (C) 3D diagrams.

Regarding SOD (PDB: 4A7G), luteolin‐7‐O‐β‐d‐glucopyranoside emerged as the most potent ligand, exhibiting multiple hydrogen bonds with key active site residues, including Asp76, Arg79, Ser105, Ser107, and His110. In addition to these conventional hydrogen bonds, this compound also engaged in pi–sigma and pi–alkyl interactions with Val103, enhancing its binding stability (Figure 3). The binding energy of luteolin‐7‐O‐β‐d‐glucopyranoside was notably high at −6.9 kcal/mol. Other phytochemicals, such as hydroxytyrosol glucoside and oleuropein, also demonstrated favorable interactions with SOD, exhibiting binding energies of −6.1 and −6.1 kcal/mol, respectively, and contributing hydrogen bonds and hydrophobic contacts at the active site.

**FIGURE 4:**
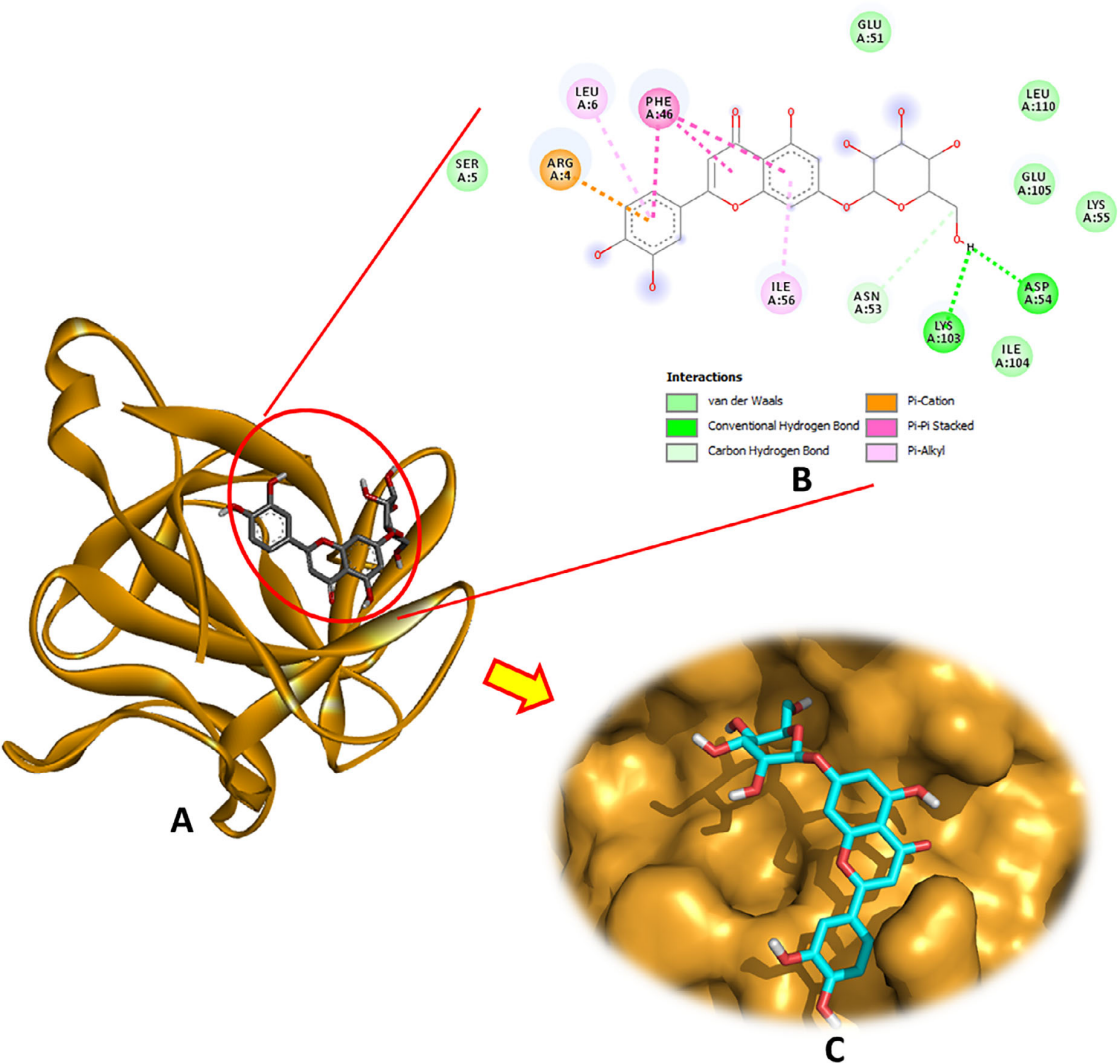
(A) Docking complex of the most bioactive compound “luteoline‐7‐O‐β‐d‐glucopyranoside” within the active site of IL‐1β (PDB: 6Y8M), (B) 2D, and (C) 3D diagrams.

Analysis of the docking interactions with IL‐1β (PDB: 6Y8M) revealed that luteolin‐7‐O‐β‐d‐glucopyranoside exhibited the strongest binding affinity among the tested phytochemicals, with a docking energy of −6.4 kcal/mol. As illustrated in Figure 4, this compound formed multiple stabilizing interactions, including two conventional hydrogen bonds with Asp54 and Lys103, a carbon–hydrogen bond with Asn53, a pi–cation interaction with Arg4, pi–pi stacking with Phe46, and pi–alkyl contacts involving Leu6 and Ile56. These interactions collectively contribute to robust and specific ligand‐receptor binding.

Oleuropein followed closely as the second most effective ligand, engaging in several key interactions, as detailed in the Supporting Information. In addition, hydroxytyrosol glucoside, secologanoside, and oleuropein aglycone formed numerous hydrogen bonds, further underscoring their promising inhibitory potential against IL‐1β.

**FIGURE 5:**
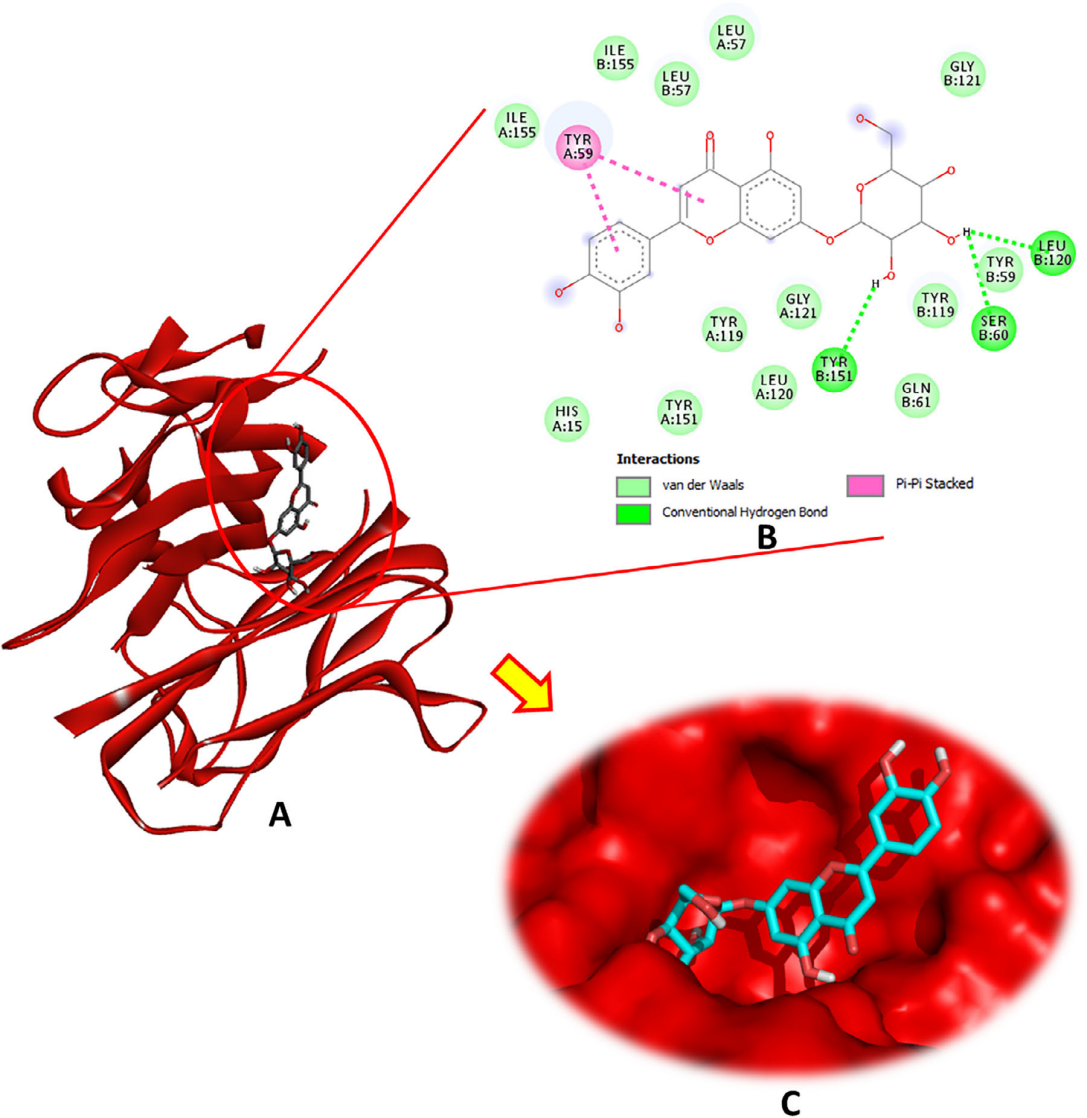
(A) Docking complex of the most bioactive compound “luteoline‐7‐O‐β‐d‐glucopyranoside” within the active site of TNF‐α (PDB: 2AZ5), (B) 2D, and (C) 3D diagrams.

Regarding the interaction with the TNF‐α receptor (PDB: 2AZ5), the docking results revealed that luteolin‐7‐O‐β‐d‐glucopyranoside demonstrated the strongest binding affinity, with a docking energy of −8.1 kcal/mol, forming three hydrogen bonds with the receptor (Figure 5). Oleuropein aglycone and secologanoside also showed significant affinities (−6.9 and −6.7 kcal/mol, respectively), each establishing four hydrogen bonds, indicative of stable interactions. Hydroxytyrosol acetate formed three hydrogen bonds with a binding energy of −5.4 kcal/mol, whereas oleuropein and hydroxytyrosol glucoside formed three and two hydrogen bonds, respectively, with energies of −7.3 and −6.6 kcal/mol.

**FIGURE 6:**
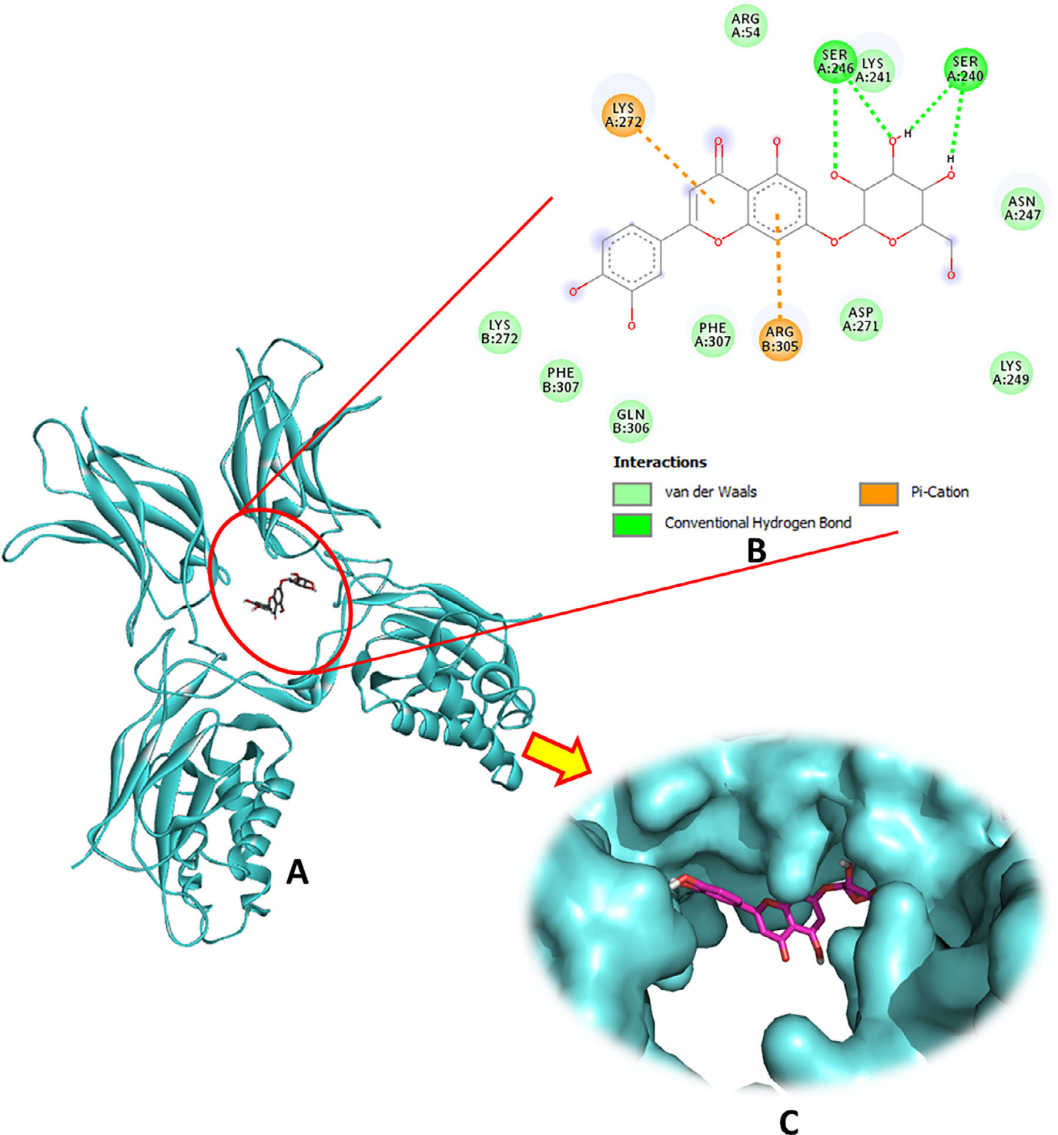
(A) Docking complex of the most bioactive compound “luteoline‐7‐O‐β‐d‐glucopyranoside” within the active site of NF‐κB (PDB: 1NFK), (B) 2D, and (C) 3D diagrams.

Regarding the interaction with NF‐κB, luteolin‐7‐O‐β‐d‐glucopyranoside exhibited the strongest binding affinity among the tested compounds, marking it as the most promising bioactive ligand. This molecule established four hydrogen bonds with the key residues Ser240 and Ser246, complemented by two significant pi–cation interactions with Lys272 and Arg305 (Figure 6). Oleuropein was the second most potent ligand, with a binding energy of −5.7 kcal/mol, forming four hydrogen bonds, as detailed in Table 1 and illustrated in Figure S6. Hydroxytyrosol glucoside ranked third, forming three hydrogen bonds, whereas secologanoside demonstrated the ability to form four hydrogen bonds, along with additional interactions, as shown in Figure S5. These binding patterns highlight the potential of these phytocompounds to effectively interact with NF‐κB and modulate its activity.

### 3.3 Protective Effects of OeLE Against Noise and Toluene‐Induced Alterations in Renal Function: Assessment of Urea and Creatinine Levels

The plasma levels of urea and creatinine were measured as sensitive indicators of renal function across all experimental groups (Table 2). In the control group, the baseline levels of urea and creatinine were 5.12 ± 0.7 mmol/L and 51.20 ± 4 µmol/L, respectively. Exposure to noise alone (Group N) led to a significant increase in both parameters, with urea reaching 6.20 ± 0.12 mmol/L and creatinine 54.31 ± 2 µmol/L (*p* < 0.05 vs. control group). These elevations indicate early signs of renal stress, likely triggered by noise‐induced oxidative and neuroendocrine imbalances, which can impair renal perfusion and glomerular filtration.

**TABLE 2:** Renal function parameters across experimental groups.

	Urea (mmol/L)	Creatinine (µmol/L)
Group C	5.12 ± 0.7	51.20 ± 4
Group OeLE	5.26 ± 0.4	50.13 ± 2
Group N	6.20 ± 0.12^*^	54.31 ± 2^*^
Group T	6.96 ± 0.5^*^	61.45 ± 0.05^*^
Group NT	7.86 ± 0.5^*^	63.45 ± 0.05^*^
Group N + OeLE	5.53 ± 0.24	53.16 ± 4
Group T + OeLE	5.61 ± 0.31	54.45 ± 6
Group NT + OeLE	5.83 ± 0.3^*^	55.65 ± 3

*Note*: The results were expressed as mean ± SD of six animals. The different pairs of groups were compared using the Mann–Whitney *U* test.

* *p* < 0.05 versus control group.

Toluene exposure (Group T) induced a more pronounced disturbance, with urea rising to 6.96 ± 0.5 mmol/L and creatinine to 61.45 ± 0.05 µmol/L (*p* < 0.05 vs. control), reflecting a stronger nephrotoxic effect. Toluene is known to accumulate in lipid‐rich tissues and compromise tubular function through mitochondrial dysfunction and oxidative stress, which likely contributed to the observed elevation.

The most significant alterations were observed in the combined exposure group (Group NT), where urea and creatinine levels reached 7.86 ± 0.5 mmol/L and 63.45 ± 0.05 µmol/L, respectively (*p* < 0.05 vs. all other groups). This finding strongly suggests an additive or synergistic effect of noise and toluene on renal function, potentially resulting from the overlapping mechanisms of oxidative damage, inflammation, and tubular transport disruption.

### 3.4 Protective Role of OeLE Against Renal Oxidative Stress Induced by Noise and Toluene Exposure in Rats

As shown in Table 3, the renal redox balance was markedly disrupted by exposure to noise and/or toluene, as demonstrated by significant alterations in the levels of MDA, CAT, and SOD. These biomarkers were selected because of their central role in redox homeostasis. MDA serves as a quantitative index of LPO and cellular oxidative damage, whereas CAT and SOD are the first enzymatic line of defense against ROS.

**TABLE 3:** Effect of exposure to noise (85 dB (A)) and/or toluene (300 ppm) on rat kidney oxidative parameters.

	Malondialdehyde (MDA) (nmol/mg prot)	Catalase (CAT) (U/mg prot)	Superoxide dismutase (SOD) (U SOD/min/mg prot)
Group C	2.513 ± 0.055^#^	3.135 ± 0.040^#^	5.27 ± 0.214^#^
Group OeLE	2.215 ± 0.025^#^	3.105 ± 0.062^#^	5.27 ± 0.154^#^
Group N	3.92 ± 0.129^*^	5.149 ± 0.015^*^	7.361 ± 0.663^*^
Group T	5.64 ± 0.258^**^	1.039 ± 0.003^**^	2.012 ± 0.663^**^
Group NT	6.75 ± 0.138^**^	0.934 ± 0.009^**^	3.677 ± 0.489^**^
Group N + OeLE	2.69 ± 0.09^#^	3.659 ± 0.014^#^	6.091 ± 0.102^#^
Group T + OeLE	2.84 ± 0.25^#^	3.615 ± 0.006^#^	5.624 ± 0.253^#^
Group NT + OeLE	3.27 ± 0.18^#^	3.677 ± 0.01^#^	5.997 ± 0.231^#^

*Note*: Results are expressed as mean ± SD of six animals. Different pairs of groups were compared using the Mann–Whitney *U* test.

* *p* < 0.05 versus control group.

** *p* < 0.01 versus control group (*N* = 6).

^#^ *p* < 0.01 versus NT group (*N* = 6).

Regarding LPO, MDA concentrations were significantly elevated in all exposed groups compared to the control group (2.513 ± 0.055 nmol/mg protein, *p* ≤ 0.01). Noise exposure increased MDA levels to 3.92 ± 0.129 nmol/mg protein, toluene exposure caused a further increase to 5.64 ± 0.258 nmol/mg protein, and the highest MDA level was recorded in the combined exposure group (6.75 ± 0.138 nmol/mg protein), highlighting a synergistic effect between both stressors. This marked increase in MDA levels, particularly under combined exposure, reflects a state of excessive LPO, likely driven by ROS overproduction and failure of antioxidant defenses.

Administration of olive leaf extract (OeLE) significantly attenuated MDA elevation, reducing the concentrations to 2.69 ± 0.09, 2.84 ± 0.25, and 3.27 ± 0.18 nmol/mg protein in the N + OeLE, T + OeLE, and NT + OeLE groups, respectively. This indicates the potent LPO inhibitory effect of OeLE under both isolated and combined oxidative insults.

CAT and SOD activities revealed differential responses to stressors. Noise exposure induced a compensatory increase in CAT (5.149 ± 0.015 U/mg protein) and SOD (7.361 ± 0.663 U/min/mg protein) levels, consistent with acute‐phase antioxidant adaptation. In contrast, toluene exposure significantly suppressed CAT (1.039 ± 0.003 U/mg protein) and SOD (2.012 ± 0.663 U/min/mg protein) activities, and the combined exposure group showed even further reductions in CAT (0.934 ± 0.009 U/mg protein) and moderate suppression of SOD (3.677 ± 0.489 U/min/mg protein). This paradoxical pattern of upregulation with noise but suppression with toluene suggests distinct mechanistic responses: noise‐induced ROS production may activate transient antioxidant responses via HPA axis stimulation, whereas toluene, due to its lipophilicity and membrane accumulation, may directly impair mitochondrial function, increase electron leakage, and inhibit antioxidant enzyme activity through catalytic damage or gene repression. The combination of both stressors appears to synergistically exhaust the antioxidant capacity, leading to redox collapse [9, 12].

Treatment with OeLE effectively restored CAT and SOD activity in all exposure groups. CAT activity returned to near‐control levels in the N + OeLE (3.659 ± 0.014 U/mg protein), T + OeLE (3.615 ± 0.006 U/mg protein), and NT + OeLE (3.677 ± 0.01 U/mg protein) groups. Similarly, SOD activity was restored to 6.091 ± 0.102, 5.624 ± 0.253, and 5.997 ± 0.231 U/min/mg of protein in the respective groups. These findings suggest that OeLE not only scavenges ROS but also preserves enzymatic integrity, likely through the modulation of redox‐sensitive signaling pathways.

### 3.5 Protective Effects of OeLE on Kidney Histopathology

**FIGURE 7:**
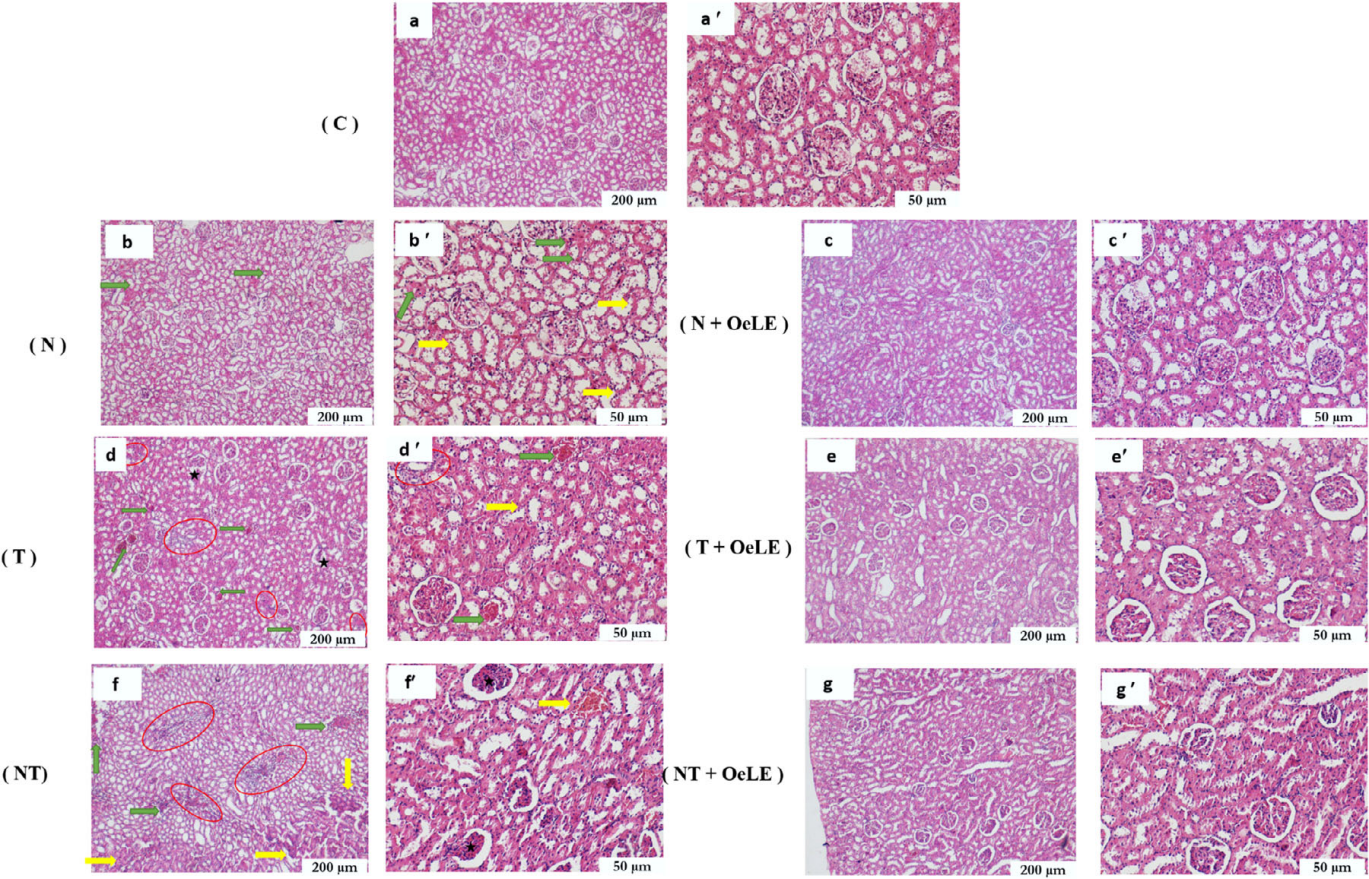
Microscopic analysis of kidney tissue (H&E): Protective effects of *Olea europaea* leaf extract (OeLE) against noise‐ and toluene‐induced histopathological alterations. (a, a′) Cross‐sections of renal tissue from the control group show normal renal architecture with well‐organized glomeruli, intact proximal and distal tubules, and no signs of inflammation or vascular congestion. (b, b′) Kidney sections from rats exposed to noise (85 dB) showing mild‐to‐moderate histological alterations, including disorganized proximal tubules with loss of brush borders (yellow arrows), epithelial cell degeneration, and vascular congestion (green arrow). (c, c′) Noise + OeLE group, in which renal morphology is largely preserved, with minimal tubular degeneration and nearly intact glomeruli, indicating protective effects against noise‐induced damage. (d, d′) Renal tissue from toluene‐exposed rats (300 ppm) displayed more severe lesions characterized by glomerular shrinkage (black star), interstitial leukocytic infiltration (red circle), and vascular congestion (green arrows). (e, e′) The toluene + OeLE group showed improved tubular integrity, reduced congestion, and minimal inflammatory infiltration, reflecting the mitigation of toluene‐induced nephrotoxicity. (f, f′) Co‐exposure to noise + toluene, exhibiting the most severe renal damage, including collapsed glomeruli with extensive glomerular cell shrinkage (black star), necrotic degeneration of proximal tubules (yellow arrows), severe interstitial inflammation (red circle), and pronounced vascular congestion (green arrows). (g, g′) Co‐exposure + OeLE group, showing partial protection with relatively preserved tubular structures and markedly reduced inflammatory infiltration, although some glomerular shrinkage persisted. a–g (×4 objective; scale bar = 200 µm); a′–g′ (×10 objective; scale bar = 50 µm).

Histopathological evaluation revealed distinct structural alterations in renal tissues across the experimental groups, reflecting the varying impacts of noise, toluene, and their combination on kidney integrity (Figure 7). In the control group (Figure 7a,a′), the renal histoarchitecture appeared normal, characterized by well‐defined glomeruli, intact tubular epithelium, and the absence of inflammatory or vascular lesions. In contrast, noise exposure alone (Figure 7b,b′) induced mild‐to‐moderate histological disturbances, including partial disorganization of the proximal tubules, loss of brush borders, epithelial cell swelling, and moderate vascular congestion. These alterations suggest early microcirculatory stress and cellular perturbations associated with sustained acoustic stimulation. The toluene‐exposed group (Figure 7d,d′) exhibited pronounced structural damage. The observed lesions included glomerular shrinkage, tubular epithelial degeneration, interstitial edema, and marked leukocytic infiltration, indicating substantial toxic injury to both the glomerular and tubular compartments. The co‐exposure group (noise + toluene) (Figure 7f,f′) exhibited the most severe renal damage. Extensive tubular necrosis, glomerular collapse, severe interstitial inflammation, and intense vascular congestion were evident, confirming the synergistic aggravation of nephrotoxic effects by the combination of physical and chemical stressors.

Remarkably, co‐administration of OeLE markedly improved renal morphology in the treated groups. In the noise + OeLE group (Figure 7c,c′), the renal parenchyma retained a near‐normal appearance with preserved glomeruli and minimal tubular alterations, indicating effective protection against noise‐induced damage. Similarly, in the toluene + OeLE group (Figure 7e,e′), the extract notably reduced vascular congestion, restored tubular epithelial organization, and minimized interstitial inflammation, highlighting its strong tissue‐preserving effects. In the combined exposure + OeLE group (Figure 7g,g′), partial but significant protection was observed. Although minor glomerular irregularities persisted, tubular architecture was largely maintained, and inflammatory infiltration was markedly diminished compared to that in the untreated co‐exposed group.

Overall, these findings demonstrate that OeLE administration effectively mitigates structural renal damage induced by both physical and chemical stressors, preserving tissue integrity and reducing inflammation. The protective effect was more pronounced under single exposure conditions but remained evident even under combined stress, supporting the robust nephroprotective potential of olive leaf extract.

## 4 Discussion

*O. europaea* (olive tree) is a medicinal plant rich in bioactive phytochemicals with potent antioxidant and anti‐inflammatory properties [2, 3]. These natural compounds play pivotal roles in protecting renal tissues from environmental and occupational insults, including physical and chemical stressors such as noise and toluene. To explore this protective potential, a comprehensive multidisciplinary study integrating in silico and in vivo approaches was conducted to assess the biochemical parameters, oxidative stress biomarkers, and histopathological alterations in the kidney tissues of the exposed rats.

Olive leaves have recently garnered considerable attention owing to their diverse pharmacological properties, including potent antioxidant, anti‐inflammatory [14, 29], antimicrobial [29], cardioprotective [16, 30], and neuroprotective effects [31]. These biological activities are primarily attributed to the high polyphenol and flavonoid content in olive leaves, which are bioactive compounds extensively recognized for their multifaceted health‐promoting potential [32, 33, 34, 35]. Flavonoids, a major subclass of polyphenols particularly abundant in olive leaves, exhibit a broad spectrum of biological effects, including potent free radical scavenging, inhibition of proinflammatory mediators, modulation of cellular signaling pathways, and protection against oxidative damage to biomolecules such as lipids, proteins, and DNA [36]. The antioxidant capacity of OeLE was corroborated through DPPH radical scavenging assays, yielding an IC_50_ value of 13.5 µg/mL, reflecting its robust ability to neutralize ROS and mitigate oxidative stress. The efficacy of these phenolic compounds is closely related to their structural characteristics, including the number, position, and conjugation of hydroxyl groups and other redox‐active moieties, which facilitate electron donation and stabilization of free radicals [37, 38]. Collectively, these properties not only rationalize the growing scientific interest in olive leaves but also highlight their potential as versatile natural agents for the prevention and management of oxidative and inflammatory injuries to tissues.

To further investigate the antioxidant potential of OeLE, we evaluated key markers of oxidative stress in renal tissue, including MDA, CAT, and SOD. Our results demonstrated that exposure to noise and/or toluene significantly disrupts renal homeostasis. Specifically, MDA levels were markedly elevated, indicating enhanced LPO, whereas CAT and SOD activities were perturbed, reflecting impairment of the enzymatic antioxidant defense system. These findings highlight the critical role of oxidative stress in mediating nephrotoxicity induced by environmental and occupational stressors. These results are consistent with those of previous studies. Abouee‐Mehrizi et al. [13] observed that co‐exposure to 100 dB white noise and 1000 ppm toluene in rabbits led to increased serum creatinine, uric acid, and albumin levels, alongside decreased urea concentrations, supporting the nephrotoxic potential of combined stressors. Similarly, prolonged exposure to high‐intensity noise has been shown to elevate creatinine and uric acid levels in rodents [39, 40].

Importantly, co‐administration of OeLE effectively countered these oxidative changes. In the NT + OeLE group, MDA concentrations were substantially reduced, and CAT and SOD activities were restored to baseline levels, demonstrating a significant protective effect. Comparable renoprotective effects were observed in the N + OeLE and T + OeLE groups, indicating that OeLE consistently mitigated oxidative damage under various stress conditions. These outcomes suggest that the antioxidant and cytoprotective properties of OeLE, largely attributed to its phenolic constituents, such as oleuropein and luteolin‐7‐O‐glucoside, play a central role in preserving renal redox balance.

The mechanistic basis for these protective effects likely involves both direct and indirect pathways. The hydroxyl‐rich structure of these polyphenols enables potent free radical scavenging, thereby intercepting ROS before they cause cellular damage [41]. In addition, these compounds may modulate endogenous antioxidant defenses, thereby enhancing the capacity of renal tissues to withstand oxidative insults. Collectively, these findings provide strong evidence that OeLE mitigates nephrotoxicity by attenuating LPO and restoring enzymatic antioxidant activity, which lays the groundwork for its observed histopathological protective effects.

The antioxidant efficacy of OeLE can be primarily attributed to its high content of bioactive phenolic compounds, particularly oleuropein and luteolin‐7‐O‐glucoside. These compounds, endowed with multiple hydroxyl groups and redox‐active moieties, exhibit potent radical‐scavenging capacity and modulate cellular antioxidant defenses by influencing enzymatic systems and redox‐sensitive signaling pathways [42, 43, 44, 45, 46]. This dual action is crucial for preserving renal cellular integrity under oxidative stress, as it prevents ROS‐mediated LPO and protein oxidation, which are central to the pathogenesis of nephrotoxicity.

In silico molecular docking studies were conducted to elucidate the molecular mechanisms underlying these protective effects. These simulations revealed strong and selective interactions between oleuropein and luteolin‐7‐O‐glucoside with key enzymatic antioxidants (SOD and CAT) and critical inflammatory mediators (IL‐1β, TNF‐α, and NF‐κB). These interactions are stabilized by a network of non‐covalent forces, including conventional hydrogen bonds, pi–pi stacking, pi–alkyl, pi–cation, and hydrophobic contacts with catalytically or structurally important residues. Such interactions influence enzyme conformation, enhance catalytic efficiency, and modulate protein dynamics, providing a mechanistic rationale for the observed in vivo antioxidant and anti‐inflammatory effects.

Notably, luteolin‐7‐O‐β‐d‐glucopyranoside consistently exhibited the highest docking scores across all targets, highlighting its central role as a multifunctional bioactive ligand. These interactions suggest the capacity to synergistically enhance endogenous antioxidant enzyme activity while concurrently inhibiting the transcriptional activation of proinflammatory cytokines, thereby orchestrating a coordinated defense against oxidative and inflammatory insults.

Collectively, these findings integrate in vivo biochemical observations, such as elevated MDA levels and perturbed CAT and SOD activities under noise and toluene exposure, with molecular insights from the docking simulations. These findings provide compelling evidence that OeLE mitigates renal oxidative stress and inflammation through direct radical scavenging and modulation of enzymatic and signaling pathways, supporting its potential as a natural nephroprotective agent against combined physical and chemical occupational stressors.

Histopathological examination revealed that exposure to noise, toluene, or their combination induced notable renal structural alterations, including tubular degeneration, glomerular shrinkage, and inflammatory cell infiltration, with the most pronounced lesions observed after the combined exposure. These findings align with those of previous studies by Abouee‐Mehrizi et al. [13], Ben Attia et al. [24], and Meydan et al. [47], who reported glomerular tuft shrinkage, tubular vacuolization, and widespread architectural disruption in toluene‐exposed animals. The convergence of these results reinforces the concept that simultaneous exposure to physical and chemical stressors exerts a synergistic nephrotoxic effect, amplifying oxidative and inflammatory damage to renal tissues.

Remarkably, supplementation with OeLE markedly preserved renal histoarchitecture. Treated rats exhibited well‐organized glomeruli, reduced tubular necrosis, and minimal inflammatory infiltration compared with untreated groups. This preservation of tissue integrity reflects the ability of the extract to counteract oxidative injury, stabilize the cellular membranes, and modulate the immune responses. The phenolic constituents of OeLE, particularly oleuropein and luteolin‐7‐O‐glucoside, appear to play a central role in attenuating structural and inflammatory deterioration, thereby maintaining renal function in adverse conditions.

When interpreted in conjunction with our in silico analyses, these histological outcomes provide compelling mechanistic support for the anti‐inflammatory and cytoprotective potential of OeLE. Molecular docking simulations demonstrated strong and selective interactions between OeLE bioactives and key proinflammatory mediators (IL‐1β, TNF‐α, and NF‐κB), suggesting a direct inhibitory effect on cytokine activation and downstream inflammatory signaling pathways. These computational insights are consistent with the observed histological protection, indicating that OeLE not only scavenges ROS but also interferes with the molecular pathways that govern inflammation.

Our findings highlight the multifaceted nephroprotective mechanisms of OeLE. In addition to its potent antioxidant capacity, the extract exerts immunomodulatory and anti‐inflammatory effects that collectively limit cytokine‐driven damage, vascular congestion, and immune cell recruitment. Such dual regulatory properties are particularly relevant in occupational and environmental contexts, where combined physical and chemical stressors synergistically disrupt renal homeostasis.

Taken together, these findings underscore the protective effect of OeLE as a natural nephroprotective agent capable of orchestrating antioxidant defenses, suppressing inflammatory cascades, and preserving renal architecture under complex multi‐stressor conditions.

## 5 Conclusion

This study demonstrated that combined exposure to noise and toluene induces severe renal dysfunction, oxidative stress, inflammation, and histopathological damage in rats. Notably, OeLE provided significant protection by restoring renal function markers, normalizing antioxidant enzyme activity, and preserving kidney structure. These effects are likely mediated by its phenolic constituents, particularly oleuropein and luteolin‐7‐O‐glucoside, through antioxidant and anti‐inflammatory mechanisms. Molecular docking further supported the interaction of these compounds with key oxidative and inflammatory targets. Overall, OeLE emerges as a promising natural nephroprotective agent against environmental stressors, including both physical and chemical insults.

### 5.1 Limitations of the Study

Although our findings provide compelling evidence for the nephroprotective and anti‐inflammatory effects of OeLE under combined noise and toluene exposure, some limitations should be acknowledged. The study did not include quantitative measurements of proinflammatory cytokines or direct assessment of NF‐κB activation, which would have strengthened the mechanistic interpretation of the observed effects at the tissue level of the study. These constraints largely reflect resource and budgetary limitations. Future studies integrating molecular, proteomic, and biochemical analyses are warranted to fully elucidate the signaling pathways involved in this phenomenon. Despite these limitations, the present study offers valuable insights that support the therapeutic potential of OeLE under complex renal stress conditions.

## Author Contributions

**Takoua Ben Attia**: conceptualization, methodology, writing – original draft, writing/review the draft. **Mabrouk Horchani**: methodology. **Mariem Salhi**: methodology. **Zeineb Dhifallah**: methodology. **Meriem Ksentini**: methodology. **Hichem Ben Jannet**: methodology. **Abada Mhamdi**: resources.

## Funding

The authors have nothing to report.

## Ethics Statement

This study was approved by the Ethics Committee of the National School of Veterinary Medicine of Sidi Thabet (Réf: 162020/FMT) and was conducted in accordance with the guidelines of the International Council for Laboratory Animal Science (ICLAS).

## Conflicts of Interest

The authors declare no conflicts of interest.

## Supporting information




**Supporting File 1**: cbdv70650‐sup‐0001‐FigureS1‐S10.docx

## Data Availability

All data and materials are available upon request.
