# Supplementary material for: Nephroprotective Potential of Olea europaea Leaf Extract Against Combined Noise and Toluene‐Induced Oxidative and Histopathological Renal Injuries in Wistar Rats
Source: Chem Biodivers. 2025 Nov 5;23(1):e02857. doi: 10.1002/cbdv.202502857 (PMC12761346; doi:10.1002/cbdv.202502857)
Supplement: Supplementary file 1 — Supporting File 1: cbdv70650‐sup‐0001‐FigureS1‐S10.docx [file CBDV-23-e02857-s001.docx]

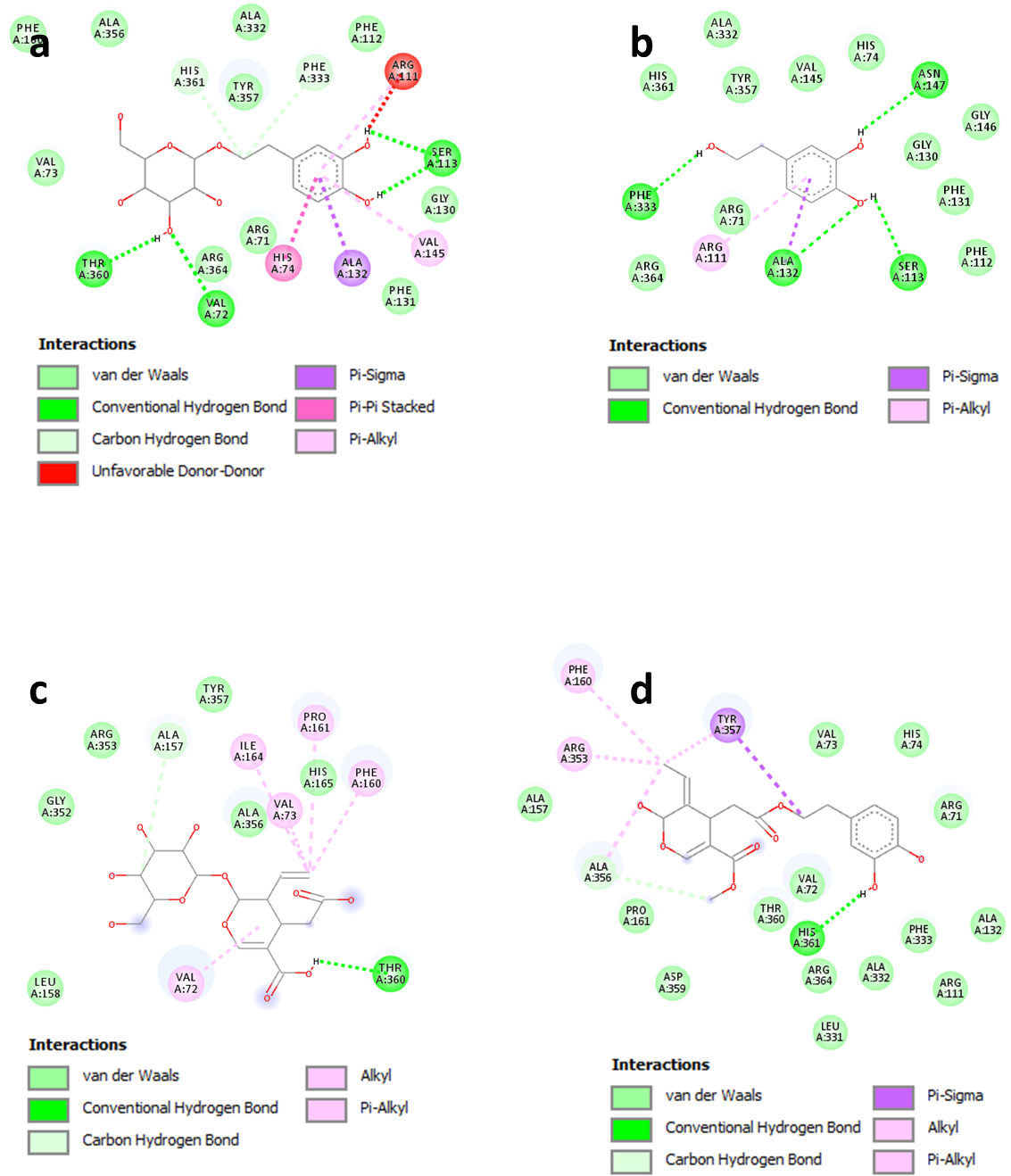


**Figure S1.** 2D interaction diagrams of (a) hydroxytyrosol glucoside, (b) hydroxytyrosol, (c) secologanoside, and (d) oleuropein aglycone within the active site of catalase (PDB: 1TGU).


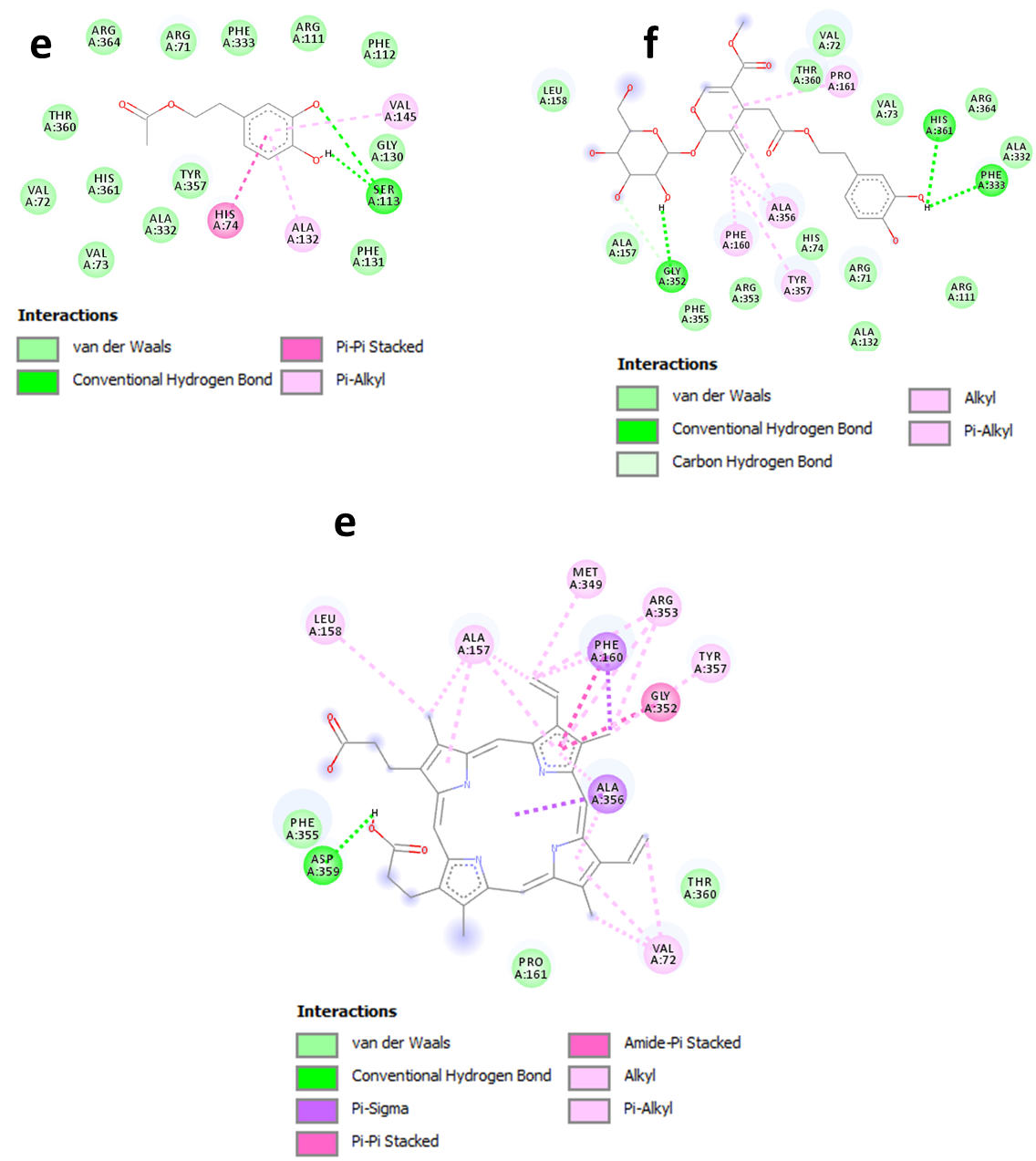


**Figure S2.** 2D interaction models of (e) hydroxytyrosol acetate, (f) oleuropein, and (g) the co-crystallized ligand within the active site of catalase (PDB: 1TGU).


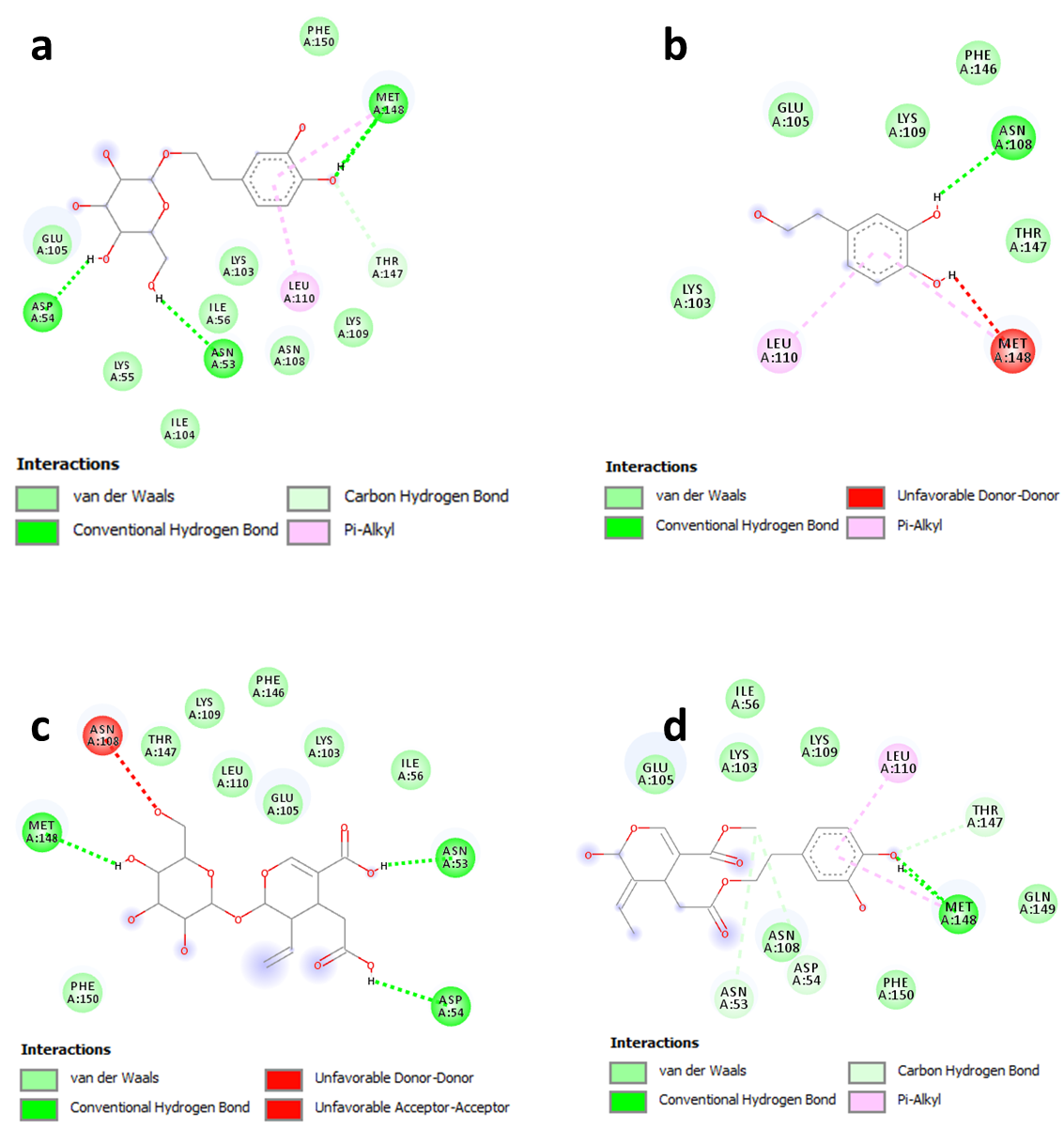


**Figure S3.** 2D interaction models of (a) hydroxytyrosol glucoside, (b) hydroxytyrosol, (c) secologanoside, and (d) oleuropein aglycone within the active site of IL-1β (PDB: 6Y8M).


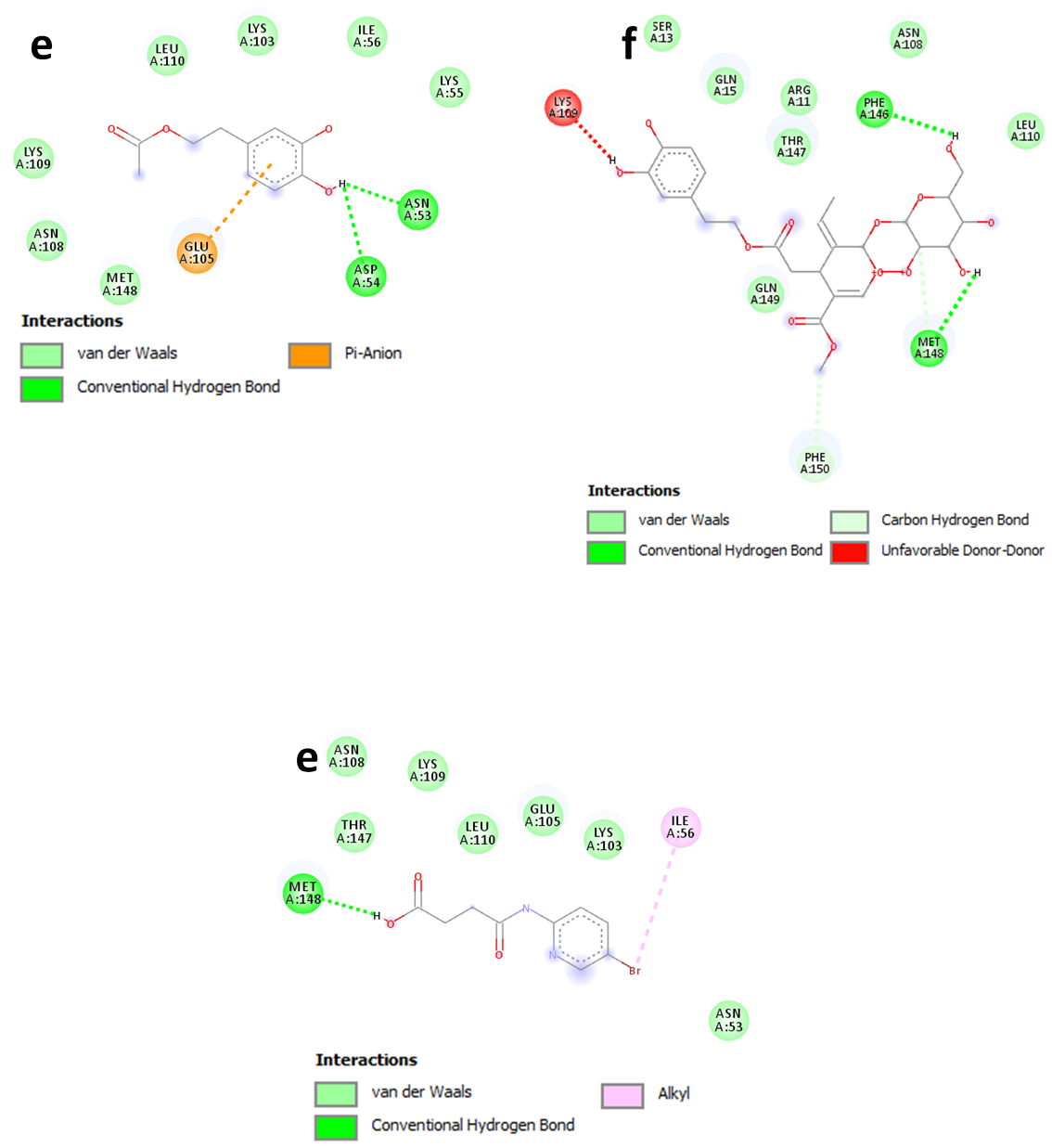


**Figure S4.** 2D interaction models of (e) hydroxytyrosol acetate, (f) oleuropein, and (g) the co-crystallized ligand within the active site of IL-1β (PDB: 6Y8M).


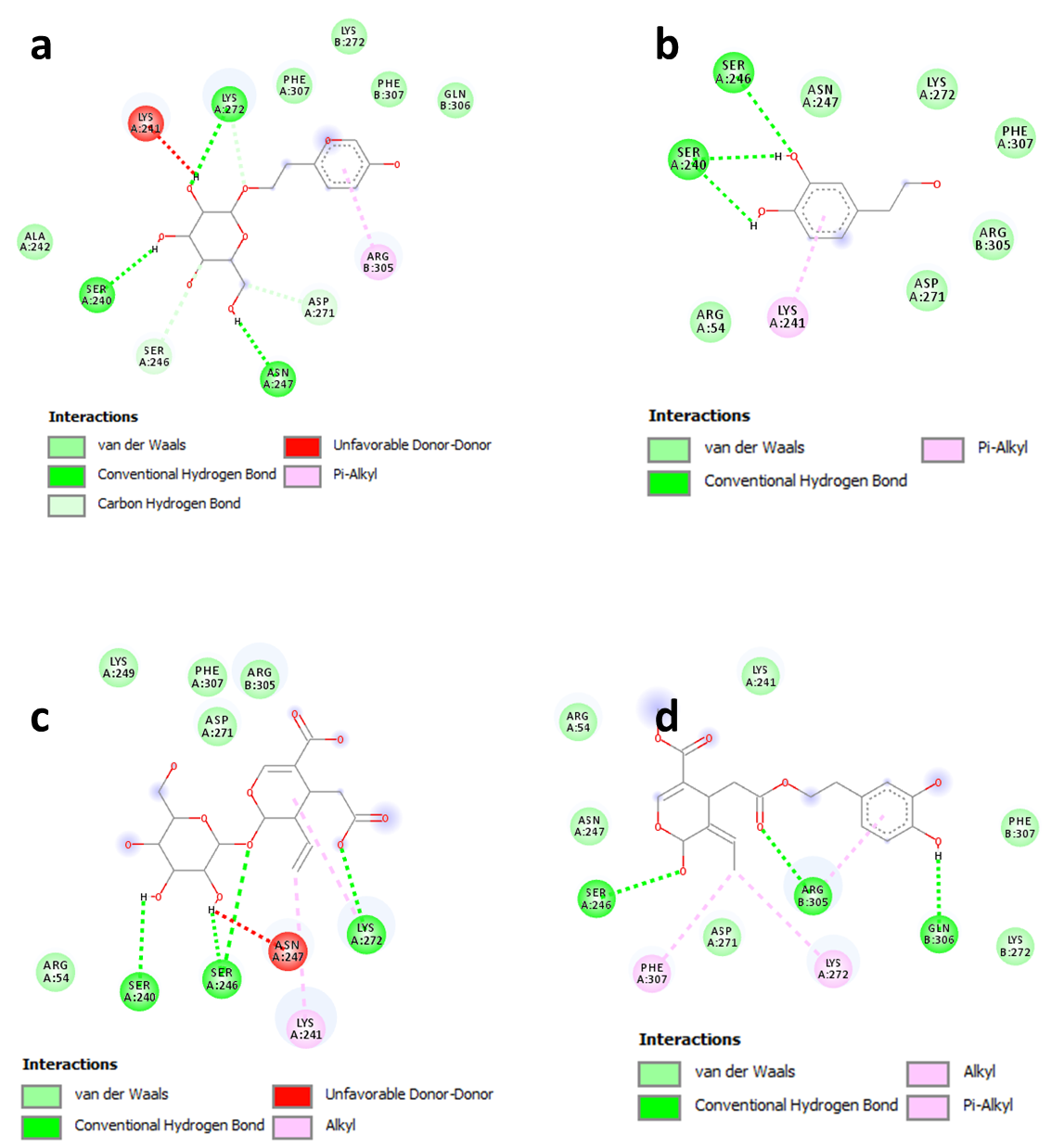


**Figure S5.** 2D interaction models of (a) hydroxytyrosol glucoside, (b) hydroxytyrosol, (c) secologanoside, and (d) oleuropein aglycone within the active site of NF-κB (PDB: 1NFK).


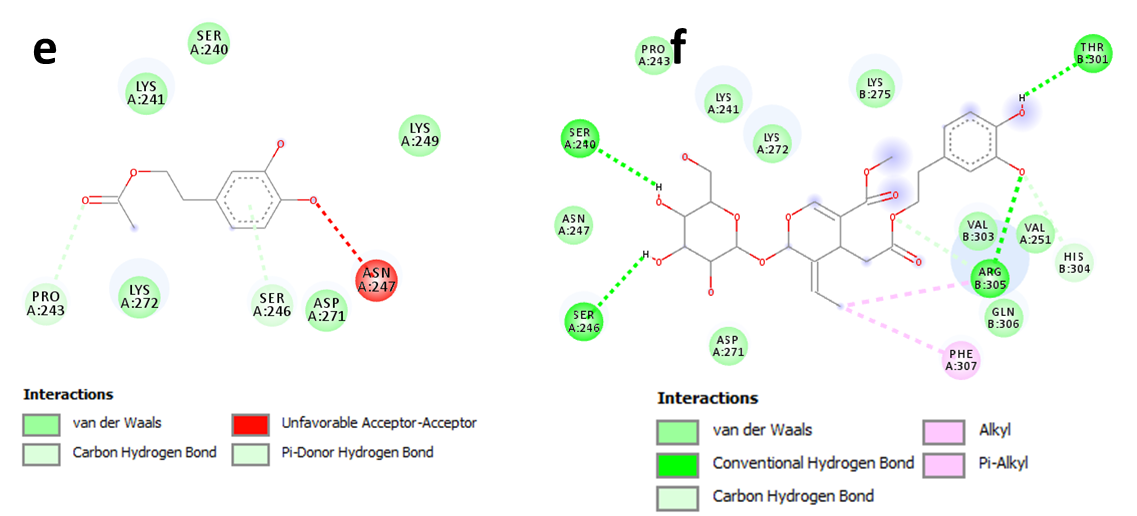


**Figure S6.** 2D interaction models of (e) hydroxytyrosol acetate and (f) oleuropein within the active site of NF-κB (PDB: 1NFK).


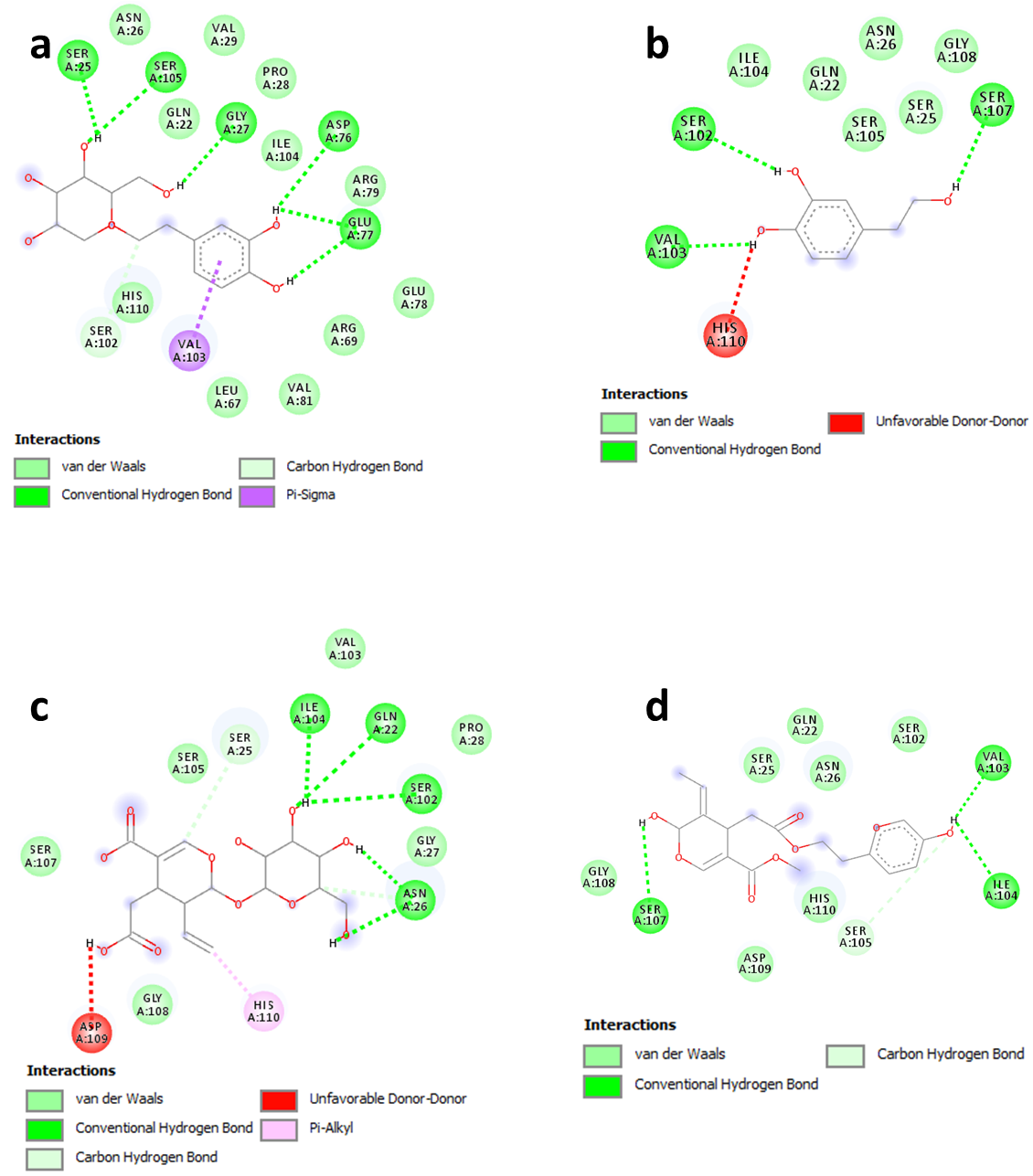


**Figure S7.** 2D interaction models of (a) hydroxytyrosol glucoside, (b) hydroxytyrosol, (c) secologanoside, and (d) oleuropein aglycone within the active site of SOD (PDB: 4A7G).


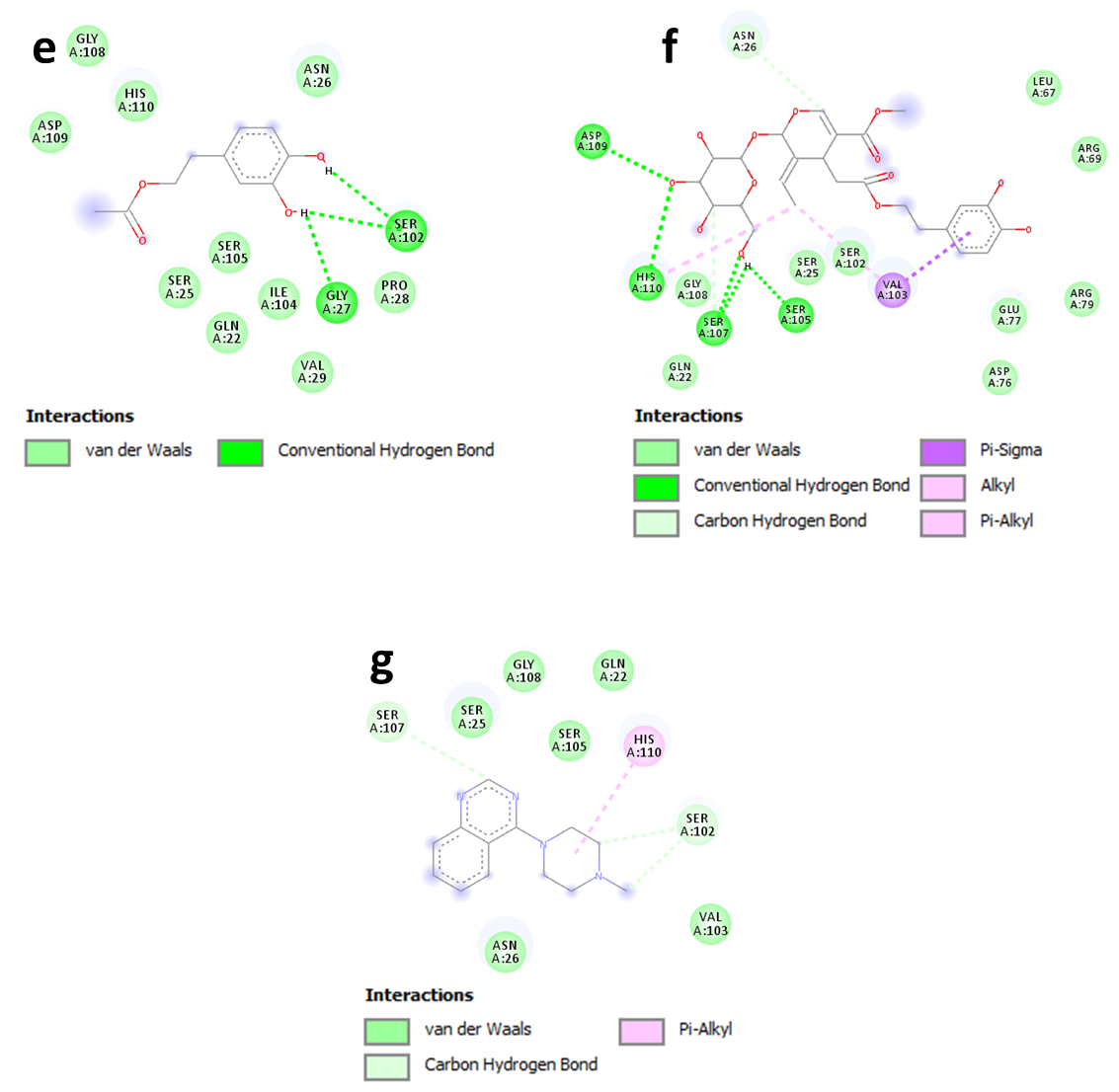


**Figure S8**. 2D interaction models of (e) hydroxytyrosol acetate, (f) oleuropein, and (g) the co-crystallized ligand within the active site of SOD (PDB: 4A7G).


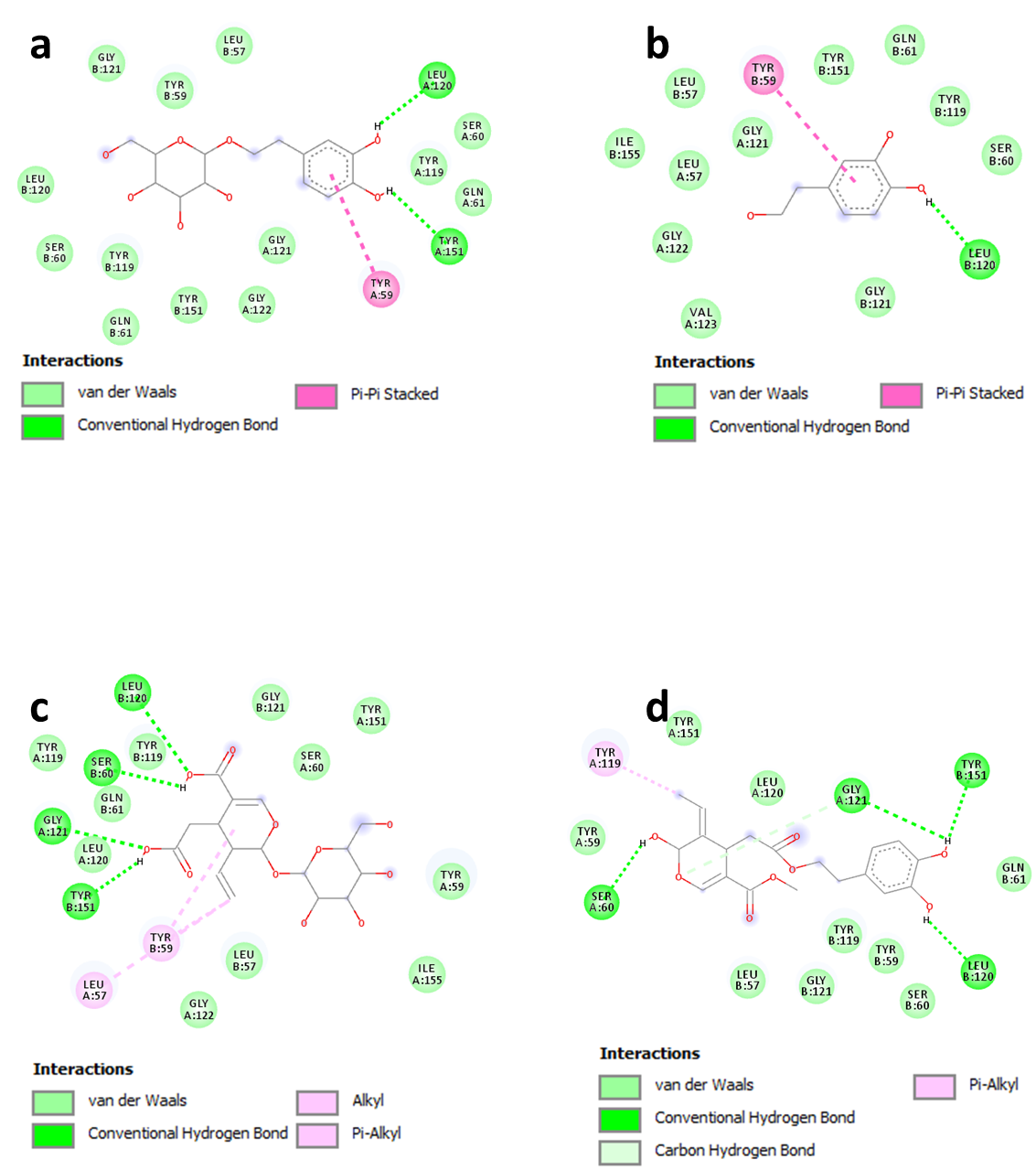


**Figure S9.** 2D interaction models of (a) hydroxytyrosol glucoside, (b) hydroxytyrosol, (c) secologanoside, and (d) oleuropein aglycone within the active site of TNF-α (PDB: 2AZ5).


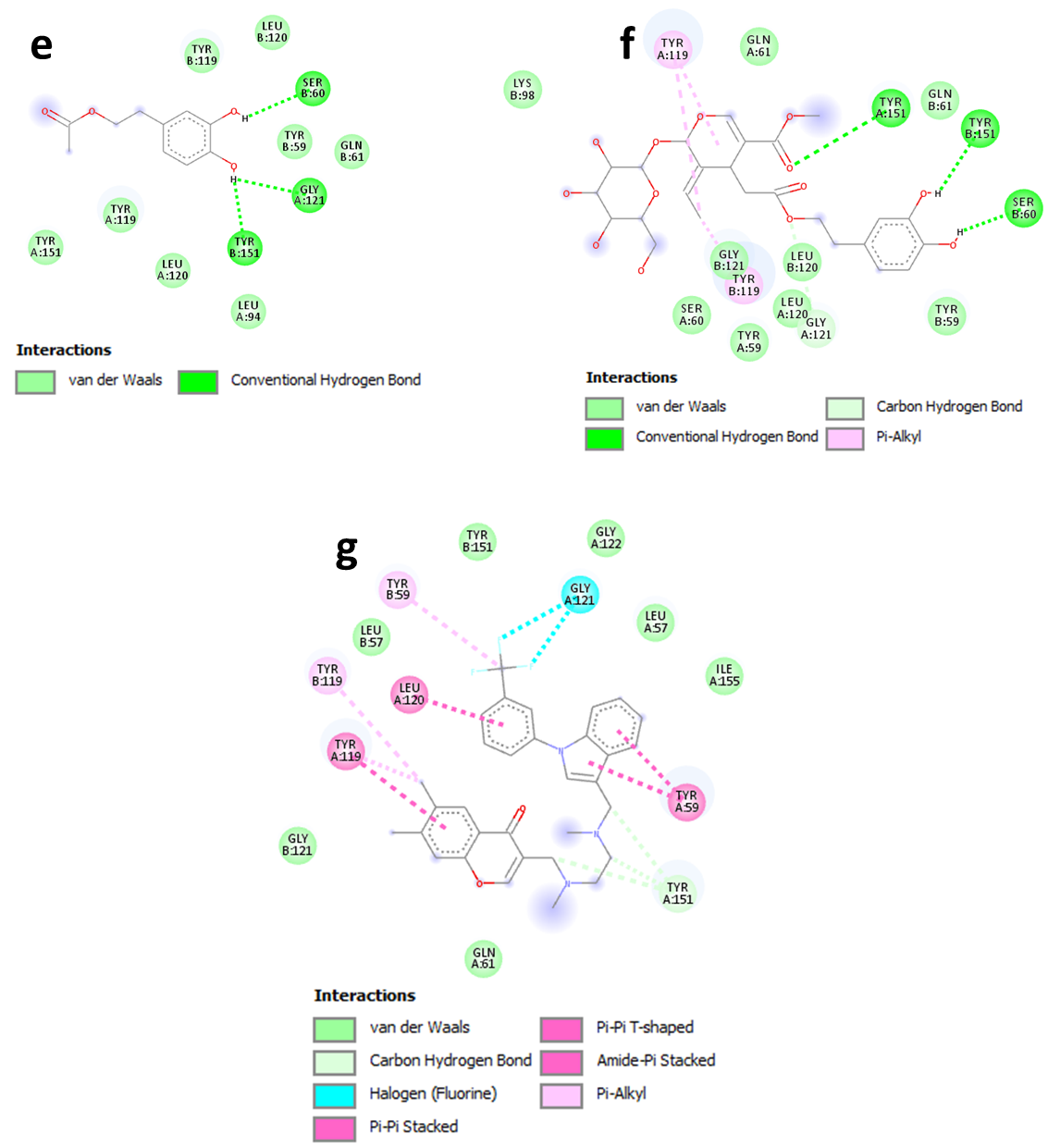


**Figure S10.** 2D interaction models of (e) hydroxytyrosol acetate, (f) oleuropein, and (g) the co-crystallized ligand within the active site of TNF-α (PDB: 2AZ5).
